# An improved reference genome for *Trifolium subterraneum* L. provides insight into molecular diversity and intra-specific phylogeny

**DOI:** 10.3389/fpls.2023.1103857

**Published:** 2023-02-15

**Authors:** Kenta Shirasawa, Roger Moraga, Andrea Ghelfi, Hideki Hirakawa, Hideki Nagasaki, Kioumars Ghamkhar, Brent A. Barrett, Andrew G. Griffiths, Sachiko N. Isobe

**Affiliations:** ^1^ Department of Frontier Research and Development, Kazusa DNA Research Institute, Kisarazu, Japan; ^2^ AgResearch, Grasslands Research Centre, Palmerston North, New Zealand; ^3^ Tea Break Bioinformatics Limited, Palmerston North, New Zealand; ^4^ Bioinformation and DDBJ Center, National Institute of Genetics, Mishima, Japan

**Keywords:** whole genome assembly, intra-specific phylogenomics, germplasm accession integrity, legume macrosynteny, subterranean clover (*Trifolium subterraneum* L.)

## Abstract

Subterranean clover (*Trifolium subterraneum* L., Ts) is a geocarpic, self-fertile annual forage legume with a compact diploid genome (n = x = 8, 544 Mb/1C). Its resilience and climate adaptivity have made it an economically important species in Mediterranean and temperate zones. Using the cultivar Daliak, we generated higher resolution sequence data, created a new genome assembly TSUd_3.0, and conducted molecular diversity analysis for copy number variant (CNV) and single-nucleotide polymorphism (SNP) among 36 cultivars. TSUd_3.0 substantively improves prior genome assemblies with new Hi-C and long-read sequence data, covering 531 Mb, containing 41,979 annotated genes and generating a 94.4% BUSCO score. Comparative genomic analysis among select members of the tribe Trifolieae indicated TSUd 3.0 corrects six assembly-error inversion/duplications and confirmed phylogenetic relationships. Its synteny with *T. pratense*, *T. repens*, *Medicago truncatula* and *Lotus japonicus* genomes were assessed, with the more distantly related *T. repens* and *M*. *truncatula* showing higher levels of co-linearity with Ts than between Ts and its close relative *T. pratense*. Resequencing of 36 cultivars discovered 7,789,537 SNPs subsequently used for genomic diversity assessment and sequence-based clustering. Heterozygosity estimates ranged from 1% to 21% within the 36 cultivars and may be influenced by admixture. Phylogenetic analysis supported subspecific genetic structure, although it indicates four or five groups, rather than the three recognized subspecies. Furthermore, there were incidences where cultivars characterized as belonging to a particular subspecies clustered with another subspecies when using genomic data. These outcomes suggest that further investigation of Ts sub-specific classification using molecular and morpho-physiological data is needed to clarify these relationships. This upgraded reference genome, complemented with comprehensive sequence diversity analysis of 36 cultivars, provides a platform for future gene functional analysis of key traits, and genome-based breeding strategies for climate adaptation and agronomic performance. Pangenome analysis, more in-depth intra-specific phylogenomic analysis using the Ts core collection, and functional genetic and genomic studies are needed to further augment knowledge of *Trifolium* genomes.

## Introduction

Legumes have a distinctive floral structure, podded fruit, and the ability by 88% of the species examined to date to form nodules with rhizobia and fix atmospheric nitrogen (Chaulagain and Frugoli, 2021). Food and feed legumes are the second most cultivated group of plants, after cereals, occupying 11% of the world’s agricultural land. They are used for food, such as soybean (*Glycine ma*x (L.) Merr.), beans (*Phaseolus vulgaris* L. or *Vicia faba* L.), and lentil (*Lens culinaris* Medik.), as well as animal feed such as lucerne (*Medicago sativa* L., Ms) and many species of clover (*Trifolium* L.).

Subterranean clover (*Trifolium subterraneum* L.; Ts) is endemic to the Mediterranean region, West Asia and the Atlantic coast of Western Europe (Morley and Katznelson, 1965; Zohary and Heller, 1984). It has been introduced successfully as a forage in many countries with similar climates including Argentina, Australia, Chile, New Zealand, South Africa, Uruguay and the USA (Nichols et al., 2013). The geocarpic ability to bury its seed-containing burrs and the grazing tolerance of Ts under suboptimal environmental conditions (Nichols et al., 2013) makes it a popular pasture in countries with production zones ill-suited to perennial pasture cover. It exhibits genetic variation for methanogenic potential (Banik et al., 2013; Muir et al., 2020), enabling lower-emission farm systems. It is also a source of diversity for adaptive response to different environmental conditions and stress, especially in environments where perennial pastures fail or are marginal.

Genome information enables species characterization, comparative analysis, and genetic improvement for breeding. Two Ts linkage maps have been developed using microsatellite (Ghamkhar et al., 2012) and single-nucleotide polymorphism (SNP) (Hirakawa et al., 2016) markers. Both span ~2,000 centimorgans, with SNP markers increasing map resolution. Additional resources available to improve this species or target varieties for different uses includes: a core collection of Ts germplasm providing a genetically representative subset of global material is available (Abdi et al., 2020); and a diverse range of over 40 cultivars with valuable traits for specific environments (Nichols et al., 2013).

Developing an enhanced Ts genome sequence extends the genomic resources for genus *Trifolium*. These genomic resources will be particularly important for elucidating the genetic basis of geocarpy, abiotic stress adaptation, methanogenesis, and animal nutrition. Compared with red clover (n=2x=14, *T. pratense*, Tp) and white clover (n=2x=16, *T. repens*, Tr), which are outbreeding perennials, Ts has a simpler genetic and genomic structure. It is an annual autogamous diploid (2n = 2x = 16), with a genome size of ~ 544 Mb/1C (Vižintin et al., 2006).

**Figure 1:**
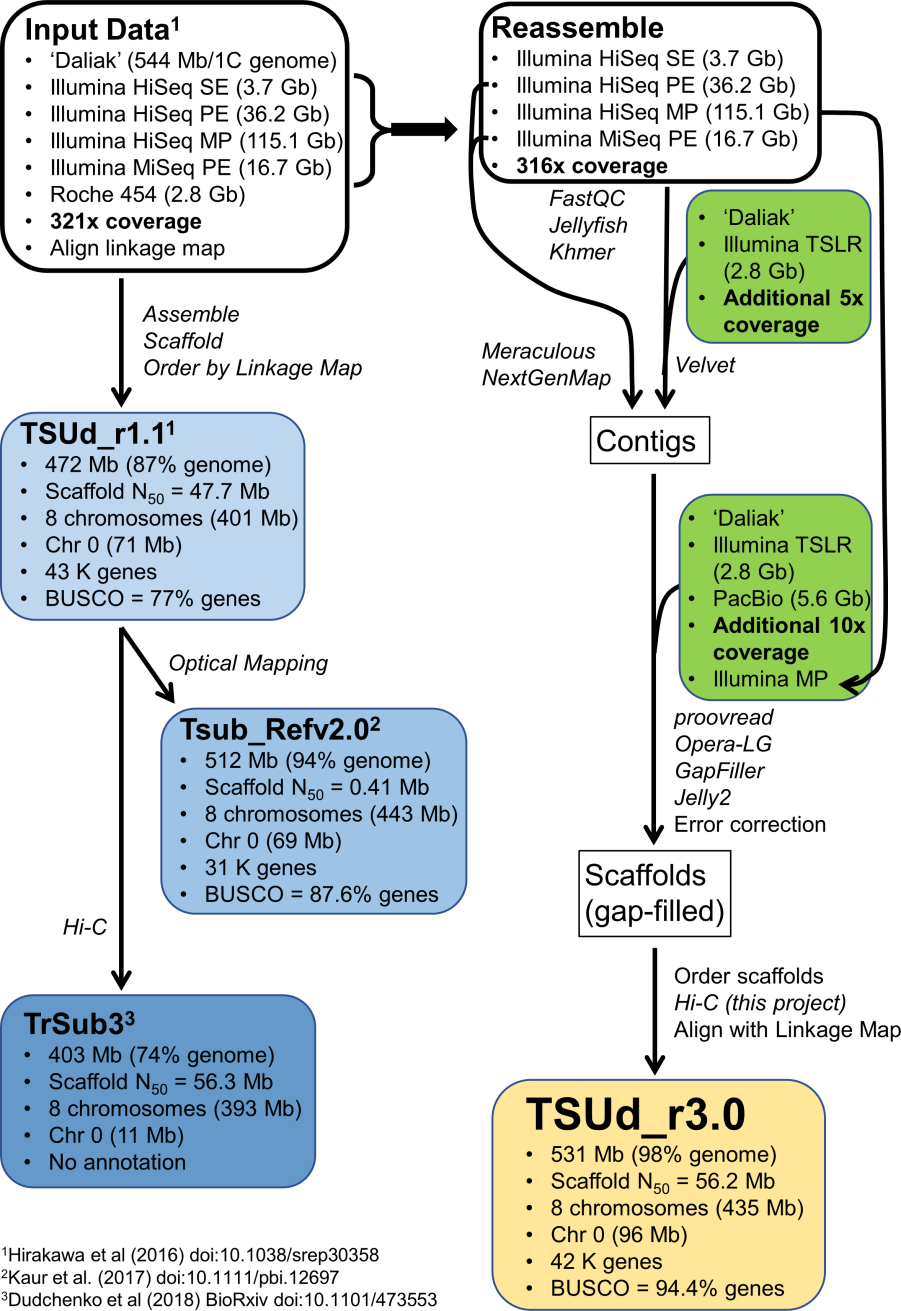
An overview showing the relationship among existing *Trifolium subterraneum* (Ts) reference genomes (TSUd_r1.1; Tsub_Refv2.0; TrSub3) and the strategy for sequencing and assembling the reference genome (TSUd_r3.0) in the current study. The percent genome encompassed by each assembly is calculated on a Ts size of 544 Mb/1C (Vižintin et al., 2006) which may differ from the value used in the various assembly publications. ‘Daliak’ = Ts cultivar used for developing the reference genome; BUSCO = Benchmarking Universal Single-Copy Orthologues scores to assess assembly gene coverage based on 1,440 reference genes; Chr 0 = sequence data not assigned to the eight assembled chromosomes; Gb, Gigabase; Hi-C, high-throughput chromosome conformation capture analysis; MP, Illumina mate-pair sequences; PE, Illumina paired-end sequences; SE, Illumina single-end sequences; TSLR, Illumina TruSeq Synthetic Long-read sequence.

Previous Ts reference genome assemblies showed a stepwise improvement in quality metrics as a representation of the Ts genome (**Figure 1**). A draft Ts assembly (TSUd_r1.1) reported by Hirakawa et al. (2016) was based on sequence data comprising Roche 454 and Illumina HiSeq reads with a range of insert sizes, which were assembled, ordered by linkage mapping, and contained approximately 43,000 annotated genes. The assembly accounts for 90% (472 Mb) of the estimated Ts genome size of 544 Mb/1C and 401 Mb was placed into eight constructed chromosomes. Synteny reported by Hirakawa et al. (2016) included the genome sequences of the forage legume *Medicago truncatula* (Mt), Tp and *Lotus japonicus* (Lj). While a significant resource, the absence of physical mapping resources resulted in an assembly with syntenic inconsistencies. This was particularly noticeable with Mt which highlighted inversions suggesting possible genome assembly duplications in Chr2, 4, 5 and 7 of the TSUd_r1.1 Ts assembly relative to Mt (Hirakawa et al., 2016).

The next assembly, Tsub_Refv2.0 (Kaur et al., 2017b) was an extension of TSUd_r1.1 where optical mapping was used to increase scaffold size and generated a draft genome covering 94% of the genome (512 Mb) of which 443 Mb were assembled into eight chromosomes (**Figure 1**). Annotations for approximately 32,000 genes were imported from TSUd_r1.1. The assembly was used to compare two cultivars at the genome level (Yuan et al., 2018), but did not address the potential scaffold order issues in the original TSUd_r1.1 draft genome from which it was derived.

The most recent Ts draft genome (**Figure 1**), TrSub3, was developed from the original TSUd_r1.1 assembly by ordering the scaffolds using Hi-C genomic proximity analysis (Dudchenko et al., 2018). The resulting draft encompassed 72% (403 Mb) of the genome with 393 Mb assembled into eight chromosomes. This assembly is not annotated and while there was improved alignment with the Mt genome, there was a note of caution that errors in the input assembly (TSUd_r1.1) could remain in the final genome (Dudchenko et al., 2018).

The current Ts assemblies described above indicate the need for a comprehensive reference genome that can be used as a resource for dissecting key agronomic traits, providing tools for genetic improvement of Ts as well as a platform for exploration of synteny with other *Trifolium* species. Syntenic analysis with other legumes, particularly within *Trifolium*, gives insight into evolutionary journeys among species. Availability of the Tr allotetraploid genome sequence and its diploid progenitor species genomes *T. occidentale* and *T. pallescens* (Griffiths et al., 2019) and assemblies of other *Trifolium* species provide a resource for a fresh investigation of synteny within the genus. Furthermore, a comprehensive Ts reference genome is a useful tool for investigating high resolution genomic diversity among Ts cultivars, an unexplored research topic. A Ts core collection (Nichols et al., 2013; Abdi et al., 2020) comprising a representative subset of the genetic diversity of this species complex is a valuable resource for this analysis. The currently accepted taxonomy of Ts recognizes three subspecies with adaptation to different environmental, soil and climatic conditions (Ghamkhar et al., 2015). Exploring germplasm and cultivar diversity can provide a resource to investigate genome-by-environment interaction. This will enable our understanding of species adaptation to different environments, and to future climate change scenarios.

In this study, we develop and provide an improved Ts reference genome (TSUd_r3.0) based on a comprehensive assembly, scaffolding with new Hi-C data, and further refinements using new long-read sequence data. We also provide comparative phylogenomics among five pasture legumes and *Arabidopsis thaliana* (At), and genome analysis of Ts with other members of Trifolieae including, for the first time, Tr. Lastly, we provide a genomic diversity assessment among 36 and within four Ts cultivars across the three subspecies. This is based on resequencing data to give insight into among and within Ts subspecific relationships and highlight potential issues for germplasm maintenance in germplasm centres.

## Materials and methods

### Repository

Configuration files for assembly and sequence quality tools that were used beyond default parameters have been placed at https://github.com/Lanilen/Subclover_genome.

### Whole genome sequencing

Following a strategy outlined in **Figure 1**, Illumina single-end (SE), paired-end (PE) and mate-pair (MP) sequences obtained in the previous study (Hirakawa et al., 2016) were used in this study (PRJDB2012, **Supplementary Table S1**). Additional long read sequences were also generated in this study (**Supplementary Tables S1, S2**). High molecular weight cellular DNA of subterranean clover cultivar ‘Daliak’ was extracted from young leaves with a Genomic-Tip (QIAGEN, Hilden, Germany) and used for construction of TruSeq synthetic long-read (TSLR) and PacBio single molecule real-time (SMRT) sequencing libraries. The TSLR library was constructed by a TruSeq synthetic long-read DNA library prep kit (Illumina, San Diego, CA) and sequences were generated by Illumina HiSeq2000 (**Supplementary Tables S1, S2**). The derived TSLR reads were assembled though the standard TruSPADES pipeline (Bankevich and Pevzner, 2016) pipeline. The SMRT library (PacBio, CA, USA) was sequenced using PacBio RS II platform.

### Data quality control and trimming

Previously generated (Hirakawa et al., 2016) Illumina single-end, paired-end, and mate-pair data (**Supplementary Table S1**; **Figure 1**) were assessed with the FastQC software package v0.10.1 (http://www.bioinformatics.babraham.ac.uk/projects/fastqc/) (Andrews, 2010) to provide metrics of sequencing quality which informed next steps for data curation. Data were then trimmed to remove adapters. For data filtering we employed Skewer (Jiang et al., 2014), an Illumina-only read trimming and filtering tool. All Illumina short read datasets were filtered as follows: Discard reads with a mean quality lower than 20, trim ends to end quality of 30, discard reads shorter than 54bp.

Total and frequency of kmers (n=17) was counted in unassembled Illumina 180 bp insert paired-end sequence data for white clover and its progenitors using Jellyfish v2.2.0 (Marçais and Kingsford, 2011) with default parameters. The data were plotted to determine maximum read depth for each specific 17-mer, and genome size was estimated as total 17-mer number divided by peak depth.

Kmer abundance graphs for all pair-end libraries were drawn with kmergenie v1.7051 (Chikhi and Medvedev, 2013; Crusoe et al., 2015) software package. After examination of the resulting graphs, two libraries were selected from the whole genome sequence (WGS) sets (DRX016491 and DRX028980) for assembly, as these two libraries showed the cleaner distribution of kmer abundances and provided sufficient coverage for assembly.

The khmer package (Crusoe et al., 2015) was then used for *in silico* digital normalization of WGS reads based on kmer abundance. We employed a different workflow than the ones recommended by the software authors. The general pipeline described by the authors removes high coverage kmers as well as low coverage kmers. This can lead to under-representation of repeat sequences in the final assembly. While de-Bruijn graph assemblers tend to collapse repeats in high coverage contigs, many of these repeats can be properly solved. Thus, khmer was used only to filter low-abundance kmer coverage reads to reduce noise. The normalize-by-median package was used to create a hash of 31 bp kmer abundances in the paired-end and single-end Illumina WGS libraries, and this hash was subsequently used with the filter-abund module to exclude reads with median kmer coverage of two or less. This adapted method allows for a reduction in the complexity of the graph assembly without reducing the representation of high coverage sequences, such as those from transposable elements, duplicated genomic regions, or closely related paralogs. All scripts and parameters used beyond default settings are located in a github repository described above.

### Contig assembly

Assembly of paired-end data was done with two different graph-based assemblers. Initially, the best kmer was chosen based on the output from kmergenie. A kmer size of 45 was selected as it was long enough to contain a high number of unique kmers compared to shorter lengths, but still showed a clear separation between the haploid kmer coverage peak and the bottom sequencing error peak.

Initial assemblies were done using Velvet (Zerbino and Birney, 2008), employing both HiSeq and MiSeq paired-end libraries, as well as the Illumina TSLR data. Fixed insert lengths were an input for the paired-end libraries, and a low cutoff coverage of four was specified to discard low-coverage contigs based on the kmer abundance graph. Finally, the –conserveLong option was employed to conserve all contigs containing an Illumina TSLR.

A second set of assemblies was done using Meraculous 2.2.4 (Chapman et al., 2011), using HiSeq and MiSeq paired-end data, as well as the shortest mate-pair Illumina library (2kb insert length). As the software does not process reads longer than 500 bp, none of the long-read libraries were used during assembly. The program was configured to run in Haploid mode, as there was no detectable heterozygosity in the kmer graph.

The addition of a single, short insert (2 kb) mate-pair library allowed for an increase of total assembly size during the gap filling step of the Meraculous pipeline, when compared to identical runs excluding all mate-pair reads. Assembly quality was assessed based on total assembly size, N50 statistics, as well as contiguity when aligning the assembled contigs with the single-read Illumina TSLR data, and whole genome alignment between the assemblies produced by the two tools. Results from both assemblers were similar, but Meraculous produced better contiguity.

### Scaffolding

To improve the Meraculous assembly scaffolds, contigs were processed using a mix of Illumina mate-pair data and long-read sequencing data (PacBio Sequel, Illumina TSLRs).

Evaluation of mate-pair library quality was done by mapping all mate-pair reads to the assembled contigs using NextGenMap (Sedlazeck et al., 2013). Discarding non-unique aligning reads and with a minimum identity of 95%, this alignment was then used to estimate mate pair insert size, size distribution, and number of outliers. One library was discarded due to a large number of inconsistent read pairs, and the rest were deemed acceptable.

PacBio long reads were error-corrected with paired-end Illumina data using the *proovread* v2.14.0 (https://github.com/BioInf-Wuerzburg/proovread) software package (Hackl et al., 2014).

Illumina mate-pair, PacBio, and Illumina SLR data were collected and processed using the software package Opera-LG (Gao et al., 2016). For each mate-pair library, the expected number of connections (based on coverage), insert length, and insert length standard deviation (based on previously described estimates) were specified.

### Gap filling

To improve assembly completeness, a two-step gap filling procedure was employed using both short and long reads. A first round of gap filling was done using Illumina paired-end short reads and the software GapFiller (Boetzer and Pirovano, 2012) using default parameters. Results were then run through Jelly2 and a second round of gap filling was done using PacBio long reads (English et al., 2012). Parameters were adjusted for the blasr step to tweak candidates and maximum score, but otherwise used the standard protocol (https://sourceforge.net/p/pb-jelly/wiki/Home/).

### Error correction

To complete the assembly, scaffolds were corrected for errors at the nucleotide level with the use of an in-house workflow (https://github.com/Lanilen/SemHelpers). Illumina TSLRs were mapped against the assembly using LAST (Kiełbasa et al., 2011), and a recursive approach was used to verify assembly bases from reads mapped with over 99% identity down to 90% identity from the resulting minor allele frequency alignment files. The recursive process was run to ensure that any single region of the reference genome underwent error-correction based on the highest scoring Illumina TSLR-genome high-scoring pair and did so only once.

### Hi-C scaffolding

A Hi-C library was constructed from the young leaves of cv. Daliak as described (Lieberman-Aiden et al., 2009). Chromatin conformation capture data was generated using a Proximo Hi-C Plant Kit (Phase Genomics, Seattle, WA). Intact cells crosslinked by a formaldehyde solution were digested using the Sau3AI restriction enzyme. The resulting library was sequenced by Illumina NextSeq500 (**Supplementary Table S2**). The 105,525,717 paired-end (PE) reads were aligned to the previously assembled 27,474 scaffolds in TSUd_r1.1 (Hirakawa et al., 2016) by BWA (Li and Durbin, 2010). The read pairs with an unmapped mate were removed by SAMtools view using the -F 12 filtering (Li et al., 2009). Chromosome-scale scaffolds were created based on the 27,424 TSUd_r1.1 scaffolds by Proximo Hi-C genome scaffolding platform (Phase Genomics) in a method similar to that described (Bickhart et al., 2017). A linkage map of the genome (Hirakawa et al., 2016) was also used during scaffolding to prevent contigs originating from different linkage groups being placed on the same chromosome during the clustering process. Approximately 132,000 separate Proximo runs were performed to optimize the number of scaffolds and scaffold construction, in order to make the scaffolds as concordant with the observed Hi-C data as possible. Juicebox (Rao et al., 2014; Durand et al., 2016) was then used to correct scaffolding errors.

### Gene prediction and annotation

To support gene modelling, transcript sequences were obtained from leaves, roots and seedlings of cv. Daliak (**Supplementary Table S2**). cDNA libraries were constructed using a TruSeq RNA Library Prep Kit (Illumina), from which 301 nucleotide paired-end (PE) sequences were generated by Illumina MiSeq. PacBio full-length cDNA sequences (Iso-Seq) were also generated from seedlings of cv. Daliak. Quality control was performed using Rcorrector (https://github.com/mourisl/Rcorrector) (Song and Florea, 2015), and Trim Galore! (http://www.bioinformatics.babraham.ac.uk/projects/trim_galore/). FastQC v0.10.1 (Andrews, 2010) was used to examine the quality metrics of reads.

RepeatModeler v1.0.11 (Smit and Hubley, 2008-2015) was used to generate a species-specific library of repetitive sequences. USEARCH v10.0.240 (Edgar, 2010) was used to align the library output to the UniProtKB (The UniProt Consortium 2018) (taxonomy Embryophyta) to predict protein existence at evidence level 1 (Experimental evidence at protein level), level 2 (Experimental evidence at transcript level) and level 3 (Protein inferred by homology). Any repeat sequence with a significant hit to UniProtKB was removed from the RepeatModeler library to minimize the number of repetitive protein sequences included in the library for repeat masking. RepeatMasker v4.0.7 (Smit et al., 2013-2015) was used to mask repetitive genomic sequence using the RepeatModeler library.

Gene prediction was performed on the masked genome as described in the gene prediction and functional annotation flowchart (**Supplementary Figure S1**). Evidence-based gene prediction was performed with transcriptome data (RNA-Seq from MiSeq; Iso-Seq sequences) and legume peptide sequences. The RNA-Seq reads were assembled by tissue (seedling root and leaf) using a genome-guided implementation of Trinity v2.4.0 (Haas et al., 2013) after alignment to the masked subterranean clover pseudomolecules using STAR (Dobin et al., 2012). PacBio SMRT Link was used to generate high-quality (HQ) Iso-Seq reads. The known peptide sequences were obtained from the Legume Information System (https://legumeinfo.org/) for the following thirteen species: *Medicago truncatula*, *Arachis duranensis*, *Arachis hypogaea*, *Arachis ipaensis*, *Cajanus cajan*, *Cicer arietinum*, *Glycine max*, *Lotus japonicus*, *Lupinus angustifolius*, *Phaseolus vulgaris*, *Trifolium pratense*, *Vigna angularis*, and *Vigna radiata*. The Trinity-assembled RNA-Seq sequences, HQ Iso-Seq sequences and the peptide sequences of these 13 legumes were then aligned independently with the assembled subterranean clover genome by Exonerate (Slater and Birney, 2005) for homology-based gene prediction using the following parameters: –model protein2genome –showtargetgff TRUE –softmaskquery yes –query target_prot.fasta –target target_dna.fasta –score 200. Meanwhile, Meanwhile, *ab initio* gene prediction was performed by Augustus (Stanke and Morgenstern, 2005) using *Arabidopsis thaliana* as the training set. In order to remove pseudogenes, the quality of the gene sequences predicted from RNA-Seq, Iso-Seq, the legume peptide sequences and the Augustus-derived *ab initio* predictions was then assessed based on the alignment to an in-house database incorporating three major databases (OrthoDB, UniProtKB and RefSeq from Viridiplantae) using Hayai-annotation with a parameter of 40% sequence (**Supplementary Figure S1**). For the first validation step (**Supplementary Figure S1**), the filtered gene sequences were aligned to the assembled subterranean clover genome sequence again using STARlong (Dobin et al., 2012). When multiple gene sequences predicted by different approaches were aligned on the same region, the longest sequences were selected, and after this second validation step (**Supplementary Figure S1**) the final set of gene sequences was created.

Hayai-Annotation Plants v1.0.1 (Ghelfi et al., 2019) using the UniProtKB/Embriophyta database was used for gene annotation of TSUd_r3.0, TSUd_r1.1, *A. thaliana* (Araport11, https://www.araport.org/data/araport11/), *L. japonicus* (Lj3.0, http://www.kazusa.or.jp/lotus/index.html (Sato et al., 2008), *M. truncatula* (Mt4.0v2, http://www.medicagogenome.org/), and *T. pratense* TGACv2 (De Vega et al., 2015). The output tables of Hayai-Annotation Plants, GO_BP_table.csv, GO_MF_table.csv and EC_table.csv, for each species were used to generate the graphic comparing all four legume species, the former version of *T. subterraneum* (TSUd_r1.1), and *A. thaliana*. The graphics were performed using Rscript. Note that a recent high-quality assembly of the *T. pratense* genome (ARS_RCv1.1; Bickhart et al., 2022) was not used in this analysis as it has yet to be annotated.

### Macrosyntenic analysis

The predicted protein-coding gene sequences were clustered with those of *A. thaliana* (Araport11), *L. japonicus* (Lj3.0), *M. truncatula* (Mt4.0v2), *T. pratense* (TGACv2) and *T. repens* (Griffiths et al., 2019) using the CD-HIT program (Fu et al., 2012). A range of cluster sequence identity (c) and alignment length (aL) parameters were assessed. Parameters c = 0.6 and aL = 0.5 were selected to optimise clustering, and all other parameters were default values.

Macrosynteny between *T. subterraneum*, and *T. pratense*, *T. repens*, *M. truncatula*, and *L. japonicas* was investigated based on homologous translated protein sequences using BLAST searches with an E-value cut-off of 1E-100. For this macrosyntenic analysis, a recent high-quality *T. pratense* assembly (ARS_RCv1.1; Bickhart et al., 2022) was used. As *T. repens* is an allotetraploid, the two subgenomes were merged into single consensus homoeologous groups, hence eight chromosomes rather than 16. Synteny plots were drawn using the gnuplot program (http://www.gnuplot.info). To create the Circos diagrams, synteny blocks were constructed by whole genome alignment using LASTZ (Harris, 2007) and comprised matches >33 kb in length. These alignments were then filtered to choose best-matching regions based on maximizing total aligned base pairs from ascending/descending sorted matches. Plots were then generated using Circa (http://omgenomics.com/circa) and blocks within 100 kb windows were merged and represented by a single line.

### Molecular phylogeny

The translated protein sequences of TSUd_r3.0 were compared by OrthoMCL (Li et al., 2003) with those of *A. thaliana* (Araport11), *L. japonicus* (Lj3.0), *M. truncatula* (Mt4.0v2), *T. pratense* (TGACv2) and *T. repens* (Griffiths et al., 2019) with the parameters c = 0.6 and aL = 0.9. The single copy genes in each cluster commonly conserved among the seven species were applied to multiple alignment by MUSCLE 3.8.31 (Edgar, 2004). The indels in the aligned sequences of the single copy genes were eliminated by Gblocks 0.91b (Castresana, 2000). The sequences of conserved blocks in the single copy genes were concatenated for each species and used for construction of phylogenetic tree by Maximum-Likelihood algorithm using MEGA X 10.0.5 (Kumar et al., 2018) with the Jones-Taylor-Thornton substitution model. In this step, *A. thaliana* was selected as outgroup. According to the TIMETREE (http://www.timetree.org), the divergence time between *L. japonicus* and *M. truncatula* is estimated as 59 MYA, and this value was used for the calibration.

### Cultivar resequencing

A total of 35 Ts cultivars in addition to Daliak were used for whole-genome diversity analysis (**Supplementary Table S2**) using resequencing of a pooled sample of at least 10 individuals per accession. The materials were obtained from the Margot Forde Germplasm Centre in New Zealand. Of the 35 cultivars, 32 were resequenced using one accession whereas cvs ‘Denmark’, ‘Leura’, and ‘Coolamon’ had two different accessions each. In total, 38 Ts accessions in addition to Daliak were resequenced. Paired-end reads were generated by Illumina HiSeq2000 (101 nt) or HiSeqX (151 nt) with coverage depth ranging from 12.4 - 41.7× based on an estimated genome size of 552.4 Mb (Hirakawa et al., 2016). The reads were mapped onto the assembled *T. subterraneum* genome (TSUd_r3.0) by Bowtie2 (Langmead and Salzberg, 2012) and variant calls were performed by SAMtools v0.1.19 (Li et al., 2009) and VarScan 2.3 (Koboldt et al., 2012) as summarized in **Supplementary Table S3**. Population structure of the resequenced lines was investigated by Admixture 1.3.0 (Alexander et al., 2009). Principal component analysis (PCA) was performed by using TASSEL 5 (Bradbury et al., 2007). Nucleotide diversity (Pi) was calculated on a per-site basis with a 500 kb sliding window using VCFtools (Danecek et al., 2011), whereas Copy Number Variations (CNV), calculated per cultivar relative to Daliak, was determined using CNV-Seq (Xie and Tammi, 2009) with a 500 kb window.

## Results

### TSUd_3.0 - an improved subterranean clover genome

In this study, a new subterranean clover (*Trifolium subterraneum*; Ts) genome assembly was generated by *de novo* assembly of existing short read data with inclusion of PacBio-derived long-read and TruSeq Synthetic Long-Read (TSLR) sequence data as detailed in **Figure 1**. This assembly was enhanced by high-throughput chromosome conformation capture (Hi-C) analysis. The Hi-C analysis generated 105,525,717 paired-end 80 bp reads (**Supplementary Table S2**) and formed 8,927 scaffolds which clustered into eight chromosome-scale scaffolds with a combined length of 441.9 Mb. This represents 93.7% of the total length of the prior assembly TSUd_r1.1 (Hirakawa et al., 2016). Alignment between the Hi-C-derived assembly and TSUd_r1.1 revealed inconsistencies (**Supplementary Figure S2A**).

Our initial assembly was augmented by the addition of 895,541 TSLR (5× coverage) and 477,975 PacBio Sequel (10× coverage) reads with mean lengths of 3,225 bp and 10,671 bp, respectively (**Supplementary Tables S1, S2**). This combined sequence resource provided 331× coverage. Integrating the TSLR data with reassembled pre-existing Illumina short-read data from Hirakawa et al. (2016) generated contigs that were scaffolded using the PacBio data resulting in eight pseudomolecules after alignment with both the Hi-C clustering, described above, and a genetic linkage map (Hirakawa et al., 2016) (**Supplementary Figures S2B, C**). This improved assembly encompassed 531 Mb accounting for 97.8% of the estimated genome size (**Table 1**). A total of 80% of this was ordered into pseudomolecules with an average length of 54.4 Mb. Whole genome shotgun Illumina sequence reads representing 57× depth were mapped back to the assembly to assess genome coverage. A mapping rate of 97.7% for these reads further indicated the assemblies encompass a high proportion of the genome. The final assembly was designated TSUd_r3.0.

**Table 1:** Genome assembly statistics for the TSUd_r3.0 assembly of subterranean clover (*Trifolium subterranean*) compared with previous drafts.

	TSUd_r1.1^1^	Tsub_Refv2.0^2^	TSUd_r3.0^3^
Estimated Genome Size			
*K*-mer (*K* = 17)^4^	552,423,008	552,423,008	552,423,008
1C Genome size^5^	544,000,000	544,000,000	544,000,000
Total Assembly Length (including N)	471,834,188	512,439,000	531,039,069
Estimated Genome Coverage	86.7%	94.2%	97.6%
Assembly Metrics			
Ps 1-8 (***scaffolds in Ps-0***)	8 (***27,416***)	1,545	8 (***13,109***)
***Total Ps + scaffolds***	***27,424***	***1,545***	***13,117***
%genome in pseudomolecules (excl Ps-0)	73.9%	80.2%	80.1%
Scaffold N_50_ (bp)	47,721,588	410,493	56,229,069
Pseudomolecule Metrics			
Ps-1 length (bp)	47,645,759	49,039,259	54,345,684
Ps-2 length (bp)	63,731,624	67,952,282	60,869,117
Ps-3 length (bp)	44,866,005	53,122,998	49,046,471
Ps-4 length (bp)	56,437,177	55,565,095	56,229,069
Ps-5 length (bp)	47,721,588	56,909,348	52,082,161
Ps-6 length (bp)	49,553,705	53,633,078	53,133,211
Ps-7 length (bp)	42,658,284	46,363,611	50,781,203
Ps-8 length (bp)	48,533,994	60,596,234	58,605,038
***Total length Ps 1-8 (bp)***	*401,148,136*	*443,181,905*	*435,091,954*
*GC (GATC) content (%);* ***Gap Ratio*** *(%)*	*33.0;* ***13.7***	*33.3;* ***11.3***	*33.3;* ***7.1***
Ps-0 length (bp)	70,686,052	69,257,095	95,947,115
* Ps-0 scaffolds*	*27,416*	–	*13,109*
* Average length Ps-0 scaffolds (bp)*	–	–	*6,319*
* Ps-0 scaffold max length (bp)*	–	–	*3,018,486*
* Ps-0 scaffold min length (bp)*	*300*	*300*	*500*
* Ps-0 scaffold N_50_ length*	–	–	*259,414*
*GC (GATC) content (%);* ***Gap Ratio*** *(%)*	*34.8;* ***22.6***	–	*34.2;* ***12.8***
***Total length Ps 1-8 + 0***	***471,834,188***	***512,439,000***	***531,039,069***
Annotation Summary			
Number of predicted genes	42,706	32,333	41,979
Total length of predicted genes (bp)	47,965,017	34,758,167	55,177,719
Mean length of predicted genes (bp)	1,123	1,075	1,314
Length of genes (bp): Max; ***Min***	15,417; ***150***	15,309; ***201***	15,309; ***90***
N_50_ of predicted genes (bp)	1,548	1,437	1,767
Proportion genes ≥ 1 kb	42.1%	–	53.7%
BUSCO^6^ Scores of Assembly (based on 1,440 reference genes)			
Complete genes - total	1,108 (77.0%)	1,261 (87.6%)	1,358 (94.4%)
* Complete genes - single copy*	*983* (*68.3%*)	*1111* (*77.2%*)	*1,255* (*87.2%*)
* Complete genes - duplicated*	*125* (*8.7%*)	*150* (*10.4%*)	*103* (*7.2%*)
Fragmented genes	138 (9.6%)	59 (4.1%)	25 (*1.7%*)
Missing genes	194 (3.9%)	120 (8.3%)	57 (*3.9%*)

Ps, pseudomolecule; bp, base pairs; ^1^ Hirakawa et al., 2016; ^2^ Kaur et al., 2017a; ^3^This study.

^4^These assemblies are based on the same Illumina paired-end sequence that was used to derive the K-mer estimate of genome size.

^5^Flow cell cytometry (Bennett and Leitch, 2011) or 1C = 0.55 pg DNA; (Vižintin et al., 2006).

^6^Benchmarking Universal Single-Copy Orthologues (BUSCO) assessment (Simão et al., 2015).

Predicted genes excludes introns; length of genes is based on cDNA.

### Pseudomolecule scaffold order enhanced compared to previous assemblies

**Figure 2:**
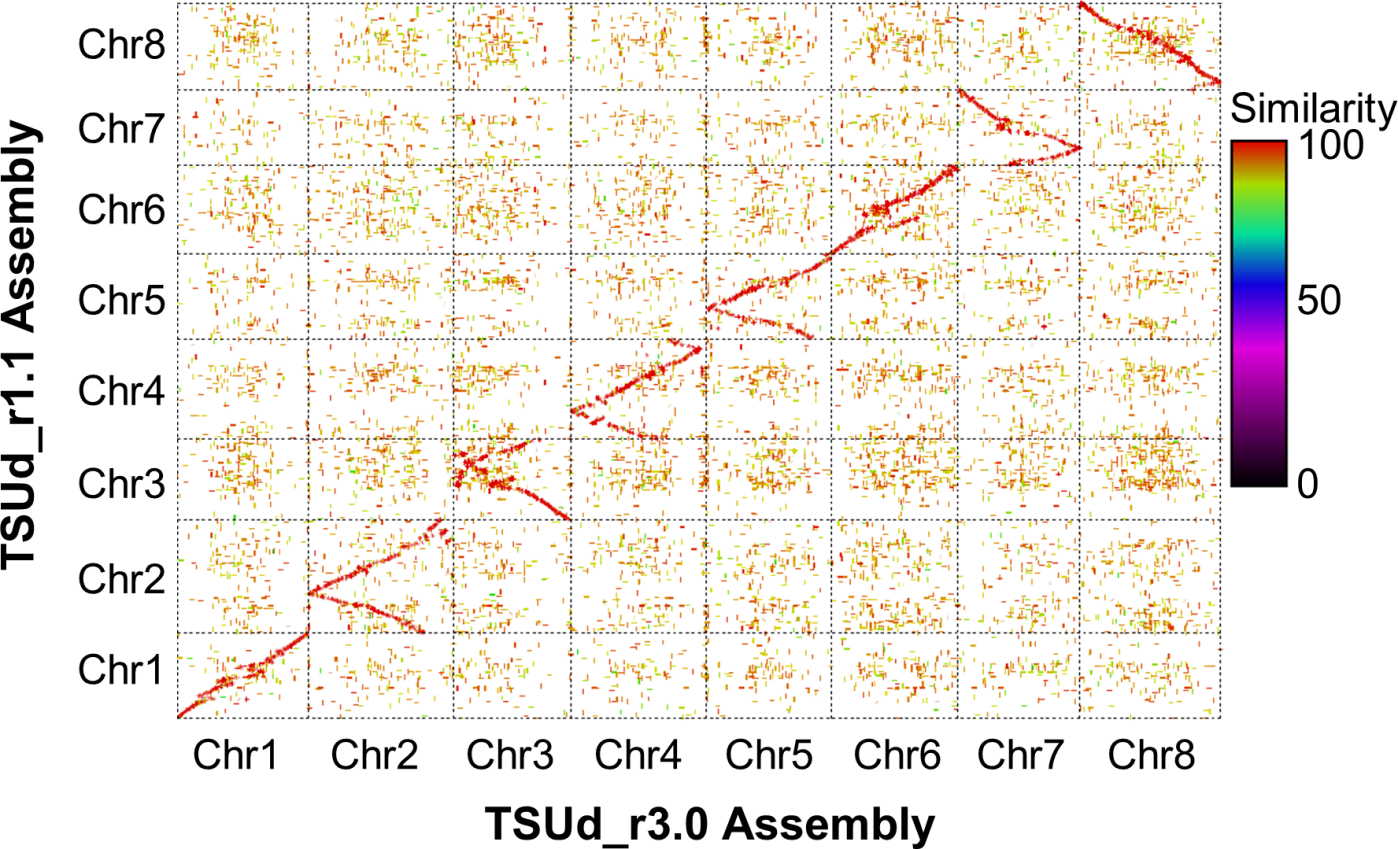
A matrix plot based on NUCmer-derived (Kurtz et al., 2004) alignment showing synteny between chromosome sequences of the original [TSUd_r1.1; (Hirakawa et al., 2016)] and current (TSUd_r3.0) subterranean clover assemblies.

**Figure 3:**
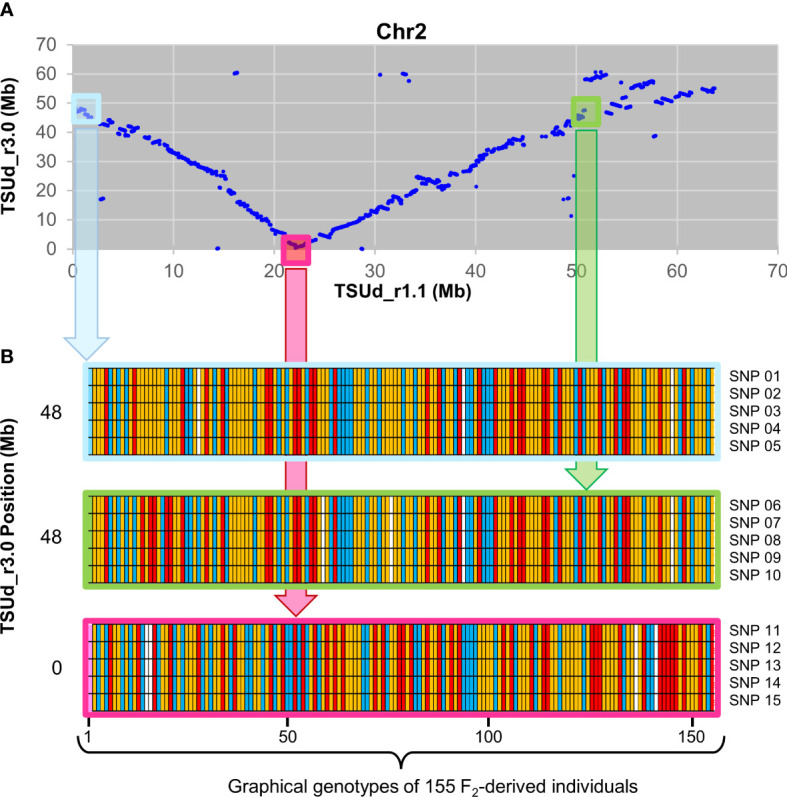
Validation of TSUd_r3.0 Chr2 assembly relative to TSUd_r1.1 using graphical genotype data from 155 F2 individuals. **(A)** Corresponding positions in megabase pairs (Mb) in chromosome 2 (Chr2) of scaffold sequences common to assemblies TSUd_r3.0 and TSUd_r1.1. The light blue and light green boxes identify scaffolds that are in close proximity in assembly TSUd_r3.0 but located at either end of Chr2 in assembly TSUd_r1.1. The light pink box denotes a Chr2 scaffold at the inflexion point of alignment of the two assemblies. **(B)** Graphical genotypes of the 155 F_2_ individuals (Hirakawa et al., 2016) of five SNPs located on each of three Chr2 scaffolds Tsud_sc00312.00 (light blue), Tsud_sc00006.10 (pink), and Tsud_sc00816.00 (light green). In the TSUd_r1.1 assembly, these scaffolds are positioned at approximately 0.8 Mb, 22.5 Mb, and 50.1 Mb, respectively. In the TSUd_r3.0 assembly, these scaffolds are positioned at approximately 48 Mb, 0 Mb and 48 Mb, respectively. The genotype at each locus for each of the 155 individuals is represented by a colored vertical bar. Red, yellow, blue and white bars represent homozygous reference allele, heterozygous (reference and alternate) and homozygous alternate allele, and missing data, respectively. SNP 01 – 05 = scaffold Tsud_sc00312.00 SNPs 10311, 15589, 164181, 164286 and 166200, respectively. SNP 06 – 10 = scaffold Tsud_sc00816.00 SNPs 136492, 144450, 28682, 29782, and 46273, respectively. SNP 11 – 15 = scaffold Tsud_sc0006.10 SNPs 126296, 155381, 164679, 180487, and 182924, respectively.

To assess relative integrity of the assembled pseudomolecules, the TSUd_r3.0 assembly was aligned with TSUd_r1.1 (**Figure 2**). Significant inversions and putative assembly duplications were identified on six of the eight pseudomolecules (Chr2, Chr3, Chr4, Chr5 Chr7 and Chr8; **Figure 2**). As the genome assemblies were constructed from individuals of the same cultivar (‘Daliak’), these structural differences suggested assembly discrepancies. The veracity of the TSUd_r3.0 assembly was investigated further using graphical genotypes of the 155 F2-derived F4 bulked DNA used for linkage map construction (Hirakawa et al., 2016). Scaffolds on Chr2 were selected based on location discrepancies when comparing the TSUd_r3.0 and TSUd_r1.1 assemblies (**Figure 3A**). A scaffold at the inflexion point in the Chr2 assembly alignment (light pink box) and two scaffolds that were adjacent in assembly TSUd_r3.0 but in different chromosome arms in the original assembly (TSUd_r1.1) (light blue and light green boxes), were analyzed for graphical genotypes.

Scaffolds in closer genomic proximity exhibit consistency of graphical genotypes for SNP loci within individuals due to the reduced likelihood of recombination. The two scaffolds identified by the light blue and green boxes in **Figure 3B** showed similar graphical genotypes for homozygosity and heterozygosity of reference and alternate alleles for each of five SNP loci per scaffold in each of the 155 genotyped individuals. This indicated these two scaffolds comprised DNA likely in close genomic proximity, supporting the placement in the current TSUd_r3.0 assembly. As expected, there was little conservation of graphical genotypes between these two scaffolds and the one identified by the light pink box. The latter was separated by ~48 Mb in assembly TSUd_r3.0, and ~20 to 30 Mb in the TSUd_r1.1 assembly (**Figure 3B**).

In summary, the analysis indicated scaffolds Tsud_sc00312.00 (light blue) and Tsud_sc00816.00 (light green) are in close genomic proximity, and in assembly TSUd­r_3.0, these two scaffold sequences were adjacent to each other at positions 47.0 to 47.6 Mb and 47.6 to 47.7 Mb, respectively. All other points of discrepancy with the previous assemblies were investigated in a similar way and the pseudomolecule structure of TSUd_r3.0 was corroborated in each case by genotype data.

### Comprehensive gene prediction and annotation

A total of 41,979 protein-coding gene sequences were predicted and annotated in assembly TSUd_r3.0 (**Table 1**), a number close to TSUd_r1.1 (42,706) and more than Tsub_Refv2.0 (32,333). Based on source data, the distribution of the best models showed that of the total predicted genes, 43% (18,051 sequences) were based on transcriptome sequence (RNA-Seq and Iso-Seq; **Supplementary Table S2**) assemblies, 30% (12,594) were homology-based supported by 13 legume species, and the remaining 27% (11,334*) ab initio* predictions. It is important to note that genes predicted by Augustus (*ab initio* prediction software) were often corroborated by the transcriptome data, however the gene prediction and annotation workflow (**Supplementary Figure S1**) prioritized the longest genes in the final validation step. Therefore, the 27% *ab initio* proportion of predicted gene models is a likely overestimate.

On average, 99.8% of the MiSeq raw reads (leaf, root, and seedling) and 82.0% of IsoSeq data (seedling) mapped to the assembly. Even though there was a high mapping rate, the transcriptome sequences accounted for approximately 43% of predicted genes. Further evidence of assembly quality and gene set completeness of TSUd_r3.0 was the marked improvement in BUSCO scores compared with previous assembly iterations (**Table 1**).

Functional gene annotation was performed using Hayai-annotation Plants (Ghelfi et al., 2019), and assembly quality further assessed by analysis of predicted gene function by assignment of Gene Ontology (GO) terms. Assembly TSUd_r3.0 had a greater proportion of predicted genes in most of the categories (~75%) within the Biological Process and Molecular Function domains, than the TSUd_r1.1 assembly (**Supplementary Figures S3A, B**). Assigning Enzyme Commission (EC) numbers to the predicted genes revealed that transferases (2.x), hydrolases (3.x) with some oxidoreductases (1.x) and isomerases (5.x) were the most common (**Supplementary Figure 3C**). When comparing across a panel of species comprising white clover (*Trifolium repens*; Tr), red clover (*T. pratense*: Tp), *Medicago truncatula* (Mt), *Lotus japonicus* (Lj) and *Arabidopsis thaliana* (At), the Trifoleae species (Ts+Tp+Tr+Mt) had similar proportions of genes in each EC class except for 3.6.4.12 (DNA helicase) where Tr was under-represented, and Mt had a greater proportion than Ts. Among the legumes, Lj generally had a greater proportion of predicted genes in each EC class, and the model plant At had an even higher proportion in most EC classes (**Supplementary Figure S3C**).

### Comparative analysis with other legume species shows fragmented alignment with red clover

Additional evidence of assembly improvement was gained from gene sequence comparisons. The set of the 41,979 predicted Ts protein-coding genes were clustered with predicted genes from a range of plant species comprising Tp, Tr, Mt, Lj and At and resulted in 34,929 Ts genes aligning with at least one other gene either in Ts or another species. Approximately 50% of the Ts genes clustered across all six species, 13% were shared within Family Leguminosae (Ts+Tp+Tr+Mt+Lj), 8% within Tribe Trifoliae (Ts+Tp+Tr+Mt) and 2% shared only among members of the genus *Trifolium* (Ts+Tp+Tr) (**Supplementary Figure S4**). While a group of 1,306 genes (4%) were shared only with the close relative Tp, and a smaller set of 799 genes (2%) only with the more distantly related Tr, less than a tenth (8%; 2,791 genes) were exclusive to Ts. Other combinations of groupings accounted for the remaining 13% of the number of clustered genes (**Supplementary Figure S4**). There was a similar pattern across these groupings when focussing on the number of gene clusters (total = 28,724 clusters) rather than clustered genes, although the percentages relative to the Ts cluster number were half those of the gene number comparisons (**Supplementary Figure S5**). Furthermore, the proportion of clusters exclusive to each species ranged from 506 (Ts) to 2,821 (Lj) with Ts having the lowest proportion of exclusive clusters (1.4%), similar to Tp (1.9%), and less than those of Tr (3.2%), Mt (4.1%), At (4.2%), and Lj (7.9%) (**Supplementary Figure S5**).

**Figure 4:**
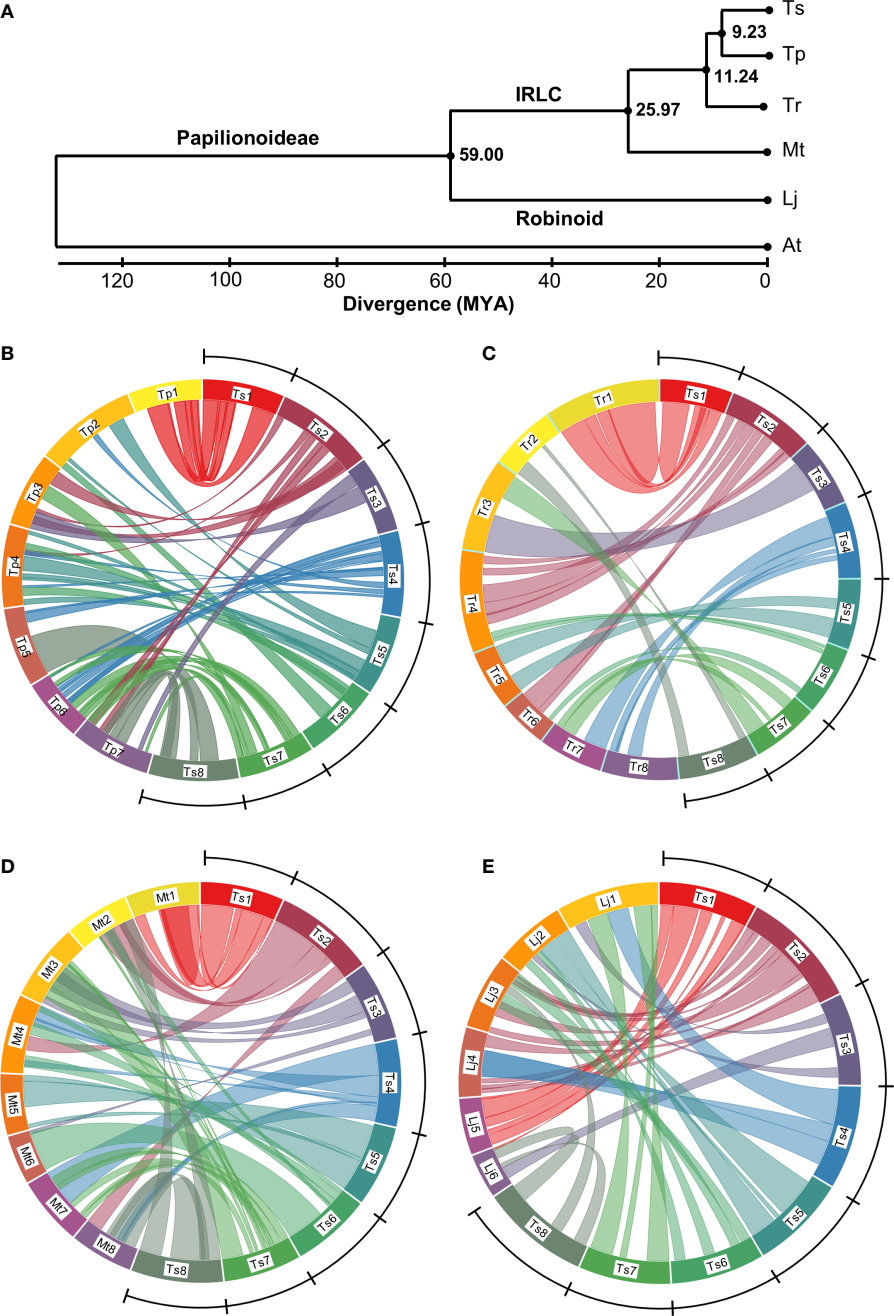
Estimated phylogeny and genome alignments among Papilionoideae legumes. **(A)** a phylogenetic tree based on 723 common single copy genes showing divergence (millions of years ago (MYA)) of subterranean clover (*Trifolium subterraneum*; Ts), red clover (*T. pratense*; Tp), white clover (*T. repens*; Tr), *Medicago truncatula* (Mt), *Lotus japonicus* (Lj) from each other and *Arabidopsis thaliana* (At) as an outgroup. IRLC identifies the inverted repeat-lacking clade of the Papilionoideae subfamily of Leguminosae which contains Vicioids such as the *Medicago* and *Trifolium* genus. **(B–E)**. Circos diagrams showing inter-chromosome relationships between the genomes of Ts and those of Tp, Tr, Mt and Lj, respectively. The ring represents pseudomolecules to scale within each circus figure. Colored lines represent synteny blocks constructed by whole genome alignment using the program LASTZ (Harris, 2007) comprising matches >33 kb in length. Blocks within 100 kb windows were merged and represented by a single line. The Ts genome is delineated by the arc and comprises chromosomes Ts1 (54 megabases (Mb)), Ts2 (61 Mb), Ts3 (49 Mb), Ts4 (56 Mbp), Ts5 (52 Mbp), Ts6 (53 Mb), Ts7 (51 Mb), Ts8 (59 Mb). The Tr genome is presented as a merged consensus of the two subgenomes into single homoeologous groups, hence eight chromosomes rather than 16.

The enhanced Ts genome assembly has provided a platform for investigating phylogenetic and macrosyntenic analysis among legumes. Focusing on 723 conserved single-copy genes, and calibrating divergence time based on the separation of Lj and Mt, the data suggest that Lj (Robinoid clade) and the inverted repeat-lacking clade containing *Medicago* and *Trifolium* genera diverged 59 million years ago (MYA), whereas the ancestors of Tr diverged from Ts and its sister clade species 11.24 MYA. Red clover, meanwhile, diverged from Ts 9.23 MYA (**Figure 4A**).

Red clover (Tp), a close relative of Ts, has seven chromosome pairs rather than the eight typical of genus *Trifolium*. Our analysis using a recent high-quality Tp genome (ARS_RCv1.1; Bickhart et al., 2022) showed numerous rearrangements between Ts and Tp, with only Chr1 maintaining substantive synteny with Ts (**Figure 4B**). Ts Chr2 showed synteny with Tp Chr3 and 7, and only half of Ts Chr3 aligned with Tp on Tp Chr3 and 7. Ts Chr4 aligned with portions of Tp Chr2, 4, 5 and 6, whereas Ts Chr 5 was syntenic with Tp Chr2 and 4. Ts Chr6 aligned with portions of Tp Chr3 and 4, whereas Ts Chr7 showed synteny with Tp Chr 3, 6 and 7. The greatest portion of Ts Chr 8 aligned with most of Tp Chr 5 and 7 (**Figure 4B**). Despite the close relationship between Ts and Tp, there were large blocks with no synteny with Tp, for example Ts Chr3, 5, 6, 7 and 8 (**Figure 4B**).

When comparing Ts with Tr, a more distant *Trifolium* relative than Tp (**Figure 4A**), there was a lack of synteny with Ts/Tr Chr3, similar to what was seen with Ts/Tp Chr3 (**Figure 4C**). There were also gaps in synteny between Ts Chr4, 5, 6, and 8 and the Tr genome. Despite the phylogenetic distance, however, Ts and Tr Chr1 had substantial synteny, and Ts Chr3 and 5 aligned with Tr Chr 3 and 5, respectively. Ts Chr2 aligned with Tr Chr 4 and 6; Ts Chr4 aligned with Tr Chr 7 and 8. With Ts Chr6, the few syntenic regions aligned with Tr Chr4 and 6. Ts Chr7 aligned with Tr Chr3 and 7, whereas the small portions of Ts/Tr synteny in Ts Chr8 aligned with Tr Chr2 (**Figure 4B**).

By contrast to the previous comparisons, Ts showed large regions of synteny across the genome with Mt, an even more distant relative than Tr (**Figure 4D**). As with the previous comparisons, Ts Chr1 aligns well with Mt Chr1, as does Ts/Mt Chr5. Ts Chr2 aligned with Mt Chr2, 4 and 8, whereas most of Ts Chr3 aligned with Mt3 except for a portion with synteny to Mt Chr6 (**Figure 4D**). Portions of Ts Chr4 align with Mt Chr4, 7 and 8. Most of Ts Chr6 aligned with Mt Chr6, although small portions showed synteny with Mt Chr2 and 4. Ts Chr7 aligned with Mt Chr7 as well as Mt Chr3 and a small section of Mt Chr2, whereas Ts Chr8 aligned with both Mt Chr2 and 8 (**Figure 4D**). The dotplot of genome alignment between Ts and Mt (**Supplementary Figure S6**) echoes the circos diagram and shows the improvement in co-linearity of the enhanced current assembly compared with TrSUd_r1.1 (Hirakawa et al., 2016). Alignment of Ts with the six chromosomes of the more distantly related Lj showed Ts Chr1, 2 and 5 exhibited syntenic conservation with Lj Chr5, 3 and 2, respectively, whereas Ts Chr4 was split between Lj Chr1 and 4 (**Figure 4E**).

### Cultivar resequencing, heterozygosity and diversity analysis

**Figure 5:**
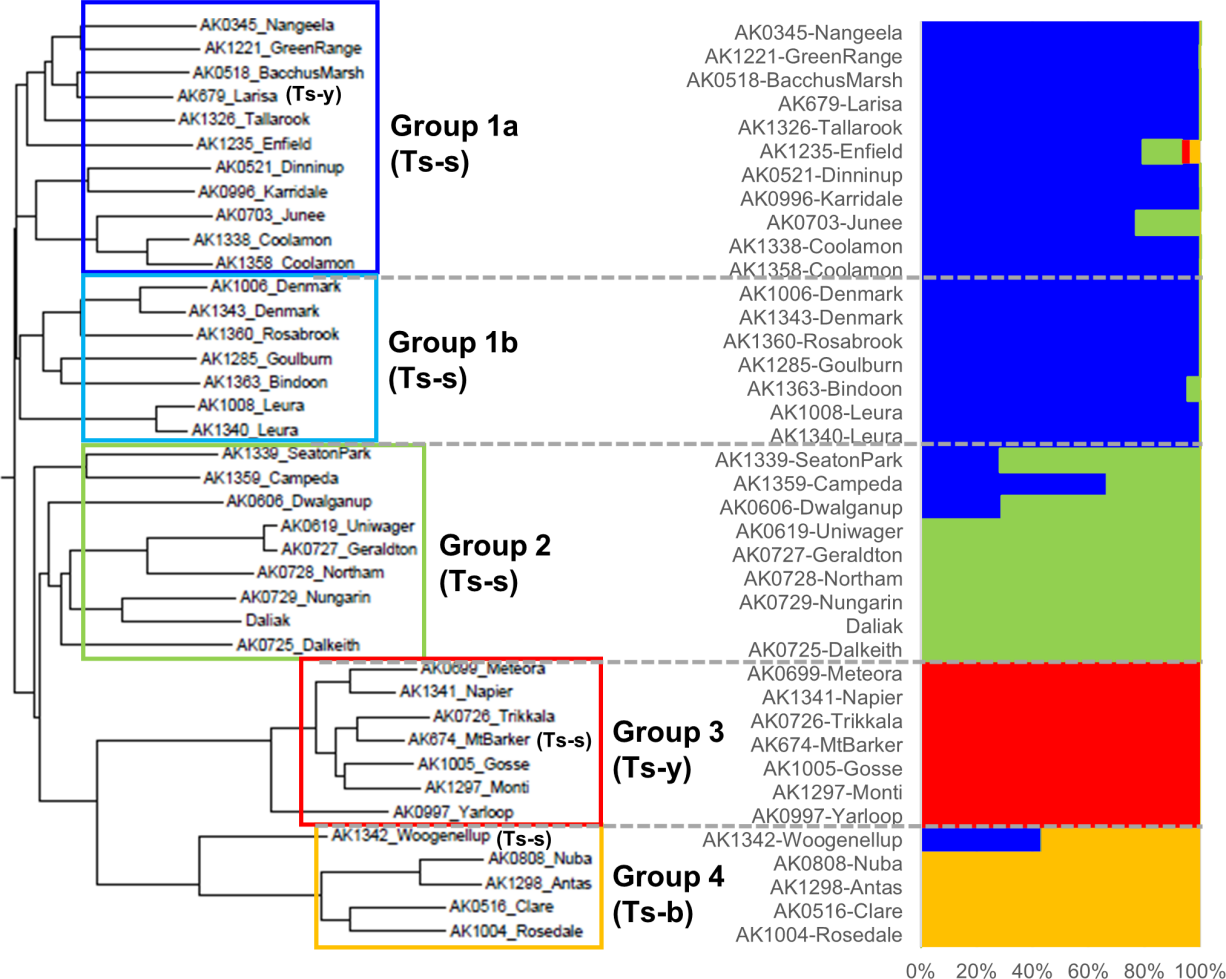
A neighbor-joining phylogenetic tree and a population structure analyzed by Admixture (K=4) of the resequenced 39 individuals representing 36 subterranean clover cultivars, three of which had multiple accessions, including ‘Daliak’ (Group 2). The AK references are identifiers of seed accessions stored in the Margot Forde Germplasm Center located at AgResearch, Palmerston North, New Zealand. The 36 cultivars were classified into the four groups and underwent PCA analysis (**Supplementary Figure S7**). Ts-s = *Trifolium subterraneum* ssp. *subterraneum*; Ts-b = *T. subterraneum* ssp. *brachycalicinum*; Ts-y = *T. subterraneum* ssp. *yanninicum*. These identifiers indicate that all individuals within the Group have been classified taxonomically as that subspecies. Exceptions are identified individually within the Group.

To explore genetic diversity, heterozygosity and copy number variation in Ts, a pool of 10 individuals from each of 35 cultivars, in addition to Daliak, was resequenced on an Illumina platform with up to 47× coverage. Three cultivars, Coolamon, Denmark and Leura, were each represented by two different accessions; therefore 38 pools were sequenced plus Daliak. Population structure based on 7,789,537 single nucleotide polymorphism (SNP) variants among the 39 pools identified four groups (K=4) (**Figure 5**, **Supplementary Table S3**). Further analysis using the Neighbor-Joining (NJ) algorithm indicated that one group comprised two closely linked sub-groups (Groups 1a and 1b; **Figure 5**) that aligned well with known pedigree. Each group was comprised predominantly of a single subspecies with *T. subterraneum* ssp. *subterraneum* placed into Groups 1a, 1b and 2, whereas *T. subterraneum* ssp. *yanninicum* and *T. subterraneum* ssp. *brachycalicinum* formed Groups 3 and 4, respectively. There were only three examples (Group 1a – Larisa; Group 3 – Mt Barker; Group 4 Woogenellup) where a cultivar classified as a particular subspecies was placed in a group comprised of another subspecies (**Figure 5**). The cultivars with multiple accessions showed dissimilarities between accessions that were greater than differences between two cultivars, such as Uniwager and Geraldton.

Principal component (PC) analysis of groups identified in the population structure analysis revealed clear differentiation by PC1 and PC2 separating Groups 3 and 4 from Groups 1 and 2 (**Supplementary Figure S7**). This supports the results from the NJ tree and structure analysis. Group 2 separated from Group 1 with PC3 whereas the Group 1 sub-groups resolved with PC4 and PC5.

**Figure 6:**
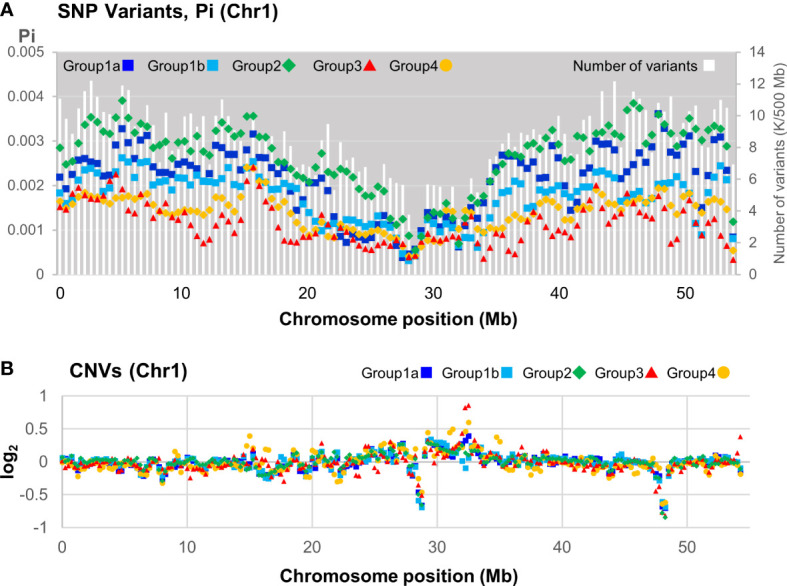
Distribution of variants along the subterranean clover chromosome 1 based on sequence data from 39 individuals representing 36 subterranean clover cultivars. **(A)** Distribution of number of variants (all) and the number of nucleotide differences per site between two sequences in a 500 kb window within a population (Pi) for each group or subgroup as defined in **Figure 5**. **(B)** Distribution of mean copy number variants (CNV) of each group or subgroup relative to Daliak in a 500 kb window.

SNP variant distribution among the 39 pools was analyzed across the genome and showed a general pattern of reduction towards the center of the pseudomolecule, indicating likely centromeric regions (**Figure 6A**; **Supplementary Figure S8A, B**). In some cases, such as Chr2, 3, and 4, there was a marked reduction in variants at the telomeric regions (**Supplementary Figure S8A**). Nucleotide diversity, calculated as Pi, was based on the number of nucleotide differences per site between two sequences within a group. This revealed Group 2 containing the reference genome Daliak was the most diverse, whereas Groups 3 and 4 consistently exhibited lower diversity (**Figure 6A**). This reflected the phylogeny and population structure analysis (**Figure 5**). Furthermore, all the groups exhibited similar levels of reduced nucleotide diversity that aligned with a reduced number of detected variants located near the putative centromere (~27 Mb Chr1; **Figure 6A**). This was consistent across pseudomolecules (**Supplementary Figure S8**). Some diversity hotspots were identified where there was a marked increase in Pi values, including positions at ~15 Mb Chr1 (**Figure 6A**), ~5 Mb and ~45 Mb Chr3 and ~48 Mb Chr 5 (**Supplementary Figure S8**).

The resequenced cultivars were also compared for CNVs relative to the Daliak reference. A distribution of mean CNV for each of the four identified groups was plotted along each chromosome (**Figure 6B**). In Chr1, there were marked reductions in CNV in the putative centromere (~27 Mb) as well as near a telomere (~48 Mb). An increase in CNV, particularly for Group 4 individuals, aligned with an increase in Pi for that group at ~15 Mb (**Figures 6A, B**). Groups 3 and 4, the two most different from the other resequenced cultivars, exhibited increased CNVs relative to the Daliak reference compared to the other groups as shown in Chr1 at positions ~10, ~15, ~18, ~32, ~35, and ~55 Mb (**Figure 6B**). Similar patterns were observed in each of the other pseudomolecules (**Supplementary Figure S9A, B**).

To gain further insight into genotype diversity within cultivars, 10 individuals each of two and three accessions from cultivars Daliak and Woogenellup, respectively, were resequenced. The 4,141 SNP variants identified relative to the Daliak reference were classed as either reference (Ref), alternate (Alt) or heterozygous (Het) genotypes and were plotted for each individual (**Supplementary Figure S10**). There were very few heterozygous loci, reflecting the species’ autogamous reproduction. As expected, the majority (95-98%) of loci in the Daliak seed lots contained reference alleles and exhibited high levels of genotype consistency among individuals. By contrast, Woogenellup had fewer reference alleles, with either ~25 or ~75% being alternate alleles. Other than accession AK1231, there was less genotype consistency in the other Woogenellup seed samples which contained individuals with either ~25 or ~75% reference alleles (**Supplementary Figure S10**).

## Discussion

We have generated a new scaffolded genome assembly for *Trifolium subterraneum* (Ts) by integrating multiple next-generation short and long genomic sequence sources combined with graphical genotypes, linkage analysis and Hi-C analysis. Revealing a genome similar in size to prior assemblies of cultivar Daliak (Kaur et al., 2017b), the new assembly TSUd_r3.0 markedly enhances pseudomolecule integrity, resolves large scale inversions, and increases the completeness of gene space. TSUd_r3.0 presents the highest quality Ts genome assembly for comparative studies with other legumes such as *T. repens* (Tr) and its progenitors (Griffiths et al., 2019), *T. pratense* (Tp) (De Vega et al., 2015; Bickhart et al., 2022), *Medicago sativa* (Ms) (Mudge and Farmer, 2021) and *M. truncatula* (Mt) (Krishnakumar et al., 2014) to date.

As described previously, earlier Ts reference genome assemblies showed a stepwise improvement in quality metrics. They were however derived from the same base assembly, TSUd_r1.1, reported by Hirakawa et al. (2016). Alignment of that assembly with a Mt genome identified inversions suggesting possible genome duplications in TSUd_r1.1 assembly relative to Mt (Figure 4A in Hirakawa et al., 2016). Alignment of our new assembly (TSUd_r3.0) with Mt did not show these inversion/duplications, yet they were detected when comparing our assembly with the original TSUd_r1.1. This strongly suggests these scaffold order issues are a feature of TRSUd_r1.1 that have been resolved in the TSUd_r3.0 reference genome. This has been supported further by graphical genotype analysis in the current study highlighting an improved scaffold order across all pseudomolecules. Comparative statistics reveal progression from earlier Ts assemblies, and that the current iteration is now of similar quality to other *Trifolium* species such as Tr (Griffiths et al., 2019) and Tp (De Vega et al., 2015; Bickhart et al., 2022). This improved Ts assembly offers a more robust platform for further genomic studies than previous assemblies. Additionally, gene prediction *via* multiple independent pathways indicated the new assembly captured the gene space more effectively than earlier iterations. This was reflected in the BUSCO score which improved from 77% (Hirakawa et al., 2016) to 87.6% (Kaur et al., 2017b) in previous iterations to the 94.4% in the current assembly. There was also a decrease in fragmented genes based on this analysis from 9.6% and 4.1% in previous versions to 1.7% in TSUd_r3.0. The transcriptome sequences alone accounted for approximately 43% of predicted genes, which contrasts with similar analysis in Tr where 86% of predicted genes were confirmed by transcriptional evidence using four diverse tissues from a mature plant (Griffiths et al., 2019). This difference in Ts may reflect sampling from a more restricted range of three tissues from seedlings, thereby yielding a transcriptome with reduced representation. However, it may reflect the gene prediction workflow which prioritized longer gene length when identical gene models were identified from the different pipelines (RNA-Seq or Iso-Seq transcriptome or peptide sequence or *ab initio*-derived) which in these cases may bias towards *ab initio* models.

Further evidence of the improved genome and annotation was the change in the number of clustered genes shared across a range of species. The current study identified 8% of 34,929 clusters as being Ts-specific compared with 49% of 30,048 clusters in the original assembly (Hirakawa et al., 2016). This striking difference in Ts-specific clusters suggests TSUd_r3.0 resolved mis-assemblies which in TSUd_r1.1 had generated an artificially large number of clusters not aligned with other species.

### Comparative genomics

Legume genomes are complex, arising from an historic whole genome duplication (Cannon et al., 2014; Ren et al., 2019). Subsequent genome evolution has resulted in x = 8 chromosomes being common within genera *Medicago* and *Trifolium*. Phylogenomic analysis based on 723 common genes in the current study indicates a common ancestor for Mt and *Trifolium* at 26 MYA, similar to the 23 MYA estimated using the Tp genome (De Vega et al., 2015), and greater than the 19 MYA calculated using an earlier iteration of the Ts genome (Hirakawa et al., 2016). This aligns with a putative origin of genus *Trifolium* in the early Miocene epoch, approximately 23 MYA (Ellison et al., 2006). Our new estimate of the divergence between *Lotus* (Robinoid) and *Medicago*/*Trifolium* spp. in the Papilionoideae inverted repeat-lacking clade (IRLC) is 59 MYA, an increase on the previous 48 MYA (Tp genome; De Vega et al., 2015) and 43 MYA (Ts genome; Hirakawa et al., 2016) estimates. Divergence of the common ancestor of Ts and the agronomically important white clover (Tr) dates back 11 MYA. Tr, however, is a recent (15-28 KYA) allopolyploid derived from the hybridization of two progenitor species that themselves diverged from a common ancestor approximately 500 KYA (Griffiths et al., 2019). This highlights that in the period since they shared common ancestors, Ts and Tr have followed quite different evolutionary paths yet retained significant macrosynteny, as described below.

More detailed analysis based on whole genome comparison is enhanced by quality assemblies. The first genome assembly of a forage legume was implemented in Mt (Young et al., 2011) and its iterative improvement as a model genome has provided a focal point for legume comparative genomic analysis. Combined with robust assemblies of Tr (Griffiths et al., 2019) and Tp (De Vega et al., 2015; Bickhart et al., 2022), our improved assembly of Ts, by correcting inversions and scaffolding errors, clarifies macrosynteny among these *Trifolium* species and Mt to a greater extent than previous Ts assemblies. The generally conserved synteny with Ts, Tr and Mt contrasts by comparison to Tp. While Ellison et al. (2006) have shown that among the economically important species of *Trifolium*, Tp is the closest relative of Ts, macrosynteny reveals a much more complex relationship. Ts and Tp belong to section Trichocephalum, whereas Tr is belongs to section Trifoliastrum within tribe Trifolieae (Ellison et al., 2006). Despite this distance in relatedness based on nuclear ribosomal DNA analysis, Ts and Tr, which are in different sections and diverged 11 MYA, have more highly conserved macrosynteny than Ts and Tp, which diverged 9 MYA. This Ts/Tr conservation includes signatures such as the inversion on the proximal arm of Chromosome 1 relative to Mt (Griffiths et al., 2013) and, as shown in the current study, approximately similar chromosome sizes. Furthermore, the Ts macrosyntenic conservation extends to large portions of the *Medicago* genome which has an earlier divergence from *Trifolium* of 26 MYA.

Presumably, the complexity of the macrosynteny between Ts and Tp was induced during the loss of a chromosome to create x = 7 in Tp. Genome transmission during chromosome loss in dysploidy is often convoluted (Mayrose and Lysak, 2021). This may have contributed to the Tp genome being reorganized relative to Ts, Tr and Mt, despite its more recent divergence time from Ts. The new genome assembly and comparison reveals rearrangement in *Trifolium* that is more complex than some other economic genera such as Gossypium and sister genera (Udall et al., 2019). With base chromosome numbers ranging from 5 to 9 (Ellison et al., 2006; Bouziane et al., 2019) and recent speciation events (Griffiths et al., 2019), the genus could be of interest for studies of genome evolution and reorganization.

### Cultivar resequencing reveals diversity and variant analysis

The impact of sequence diversity, copy number variants (CNVs) and structural variants (SVs) on species diversification and breeding outcomes has come to the fore recently (Wellenreuther et al., 2019; Della Coletta et al., 2021). Characterization of SNP diversity and CNV/SVs is a motivation for widespread resequencing for pangenome analysis and is critical to accessing molecular diversity for breeding (McCouch et al., 2020). Such surveys in Ts are of particular interest due to the broad adaptation of the species, subspecific classification, and presence of chromosome-level SVs (Bouziane et al., 2019).

Here we report the first steps toward a wider pangenome analysis for Ts, using 36 cultivars from a core collection of subterranean clover (Ghamkhar et al., 2015). Our analysis has revealed SNPs and CNVs across the genome which have given insight into intra-specific and intra-subspecific relationships. There are currently three accepted subspecies in Ts: ssp. *subterraneum*, ssp. *yanninicum*, and ssp. *brachycalycinum* (Nichols et al., 2013). While there are geographic distribution overlaps among these subspecies (Ghamkhar et al., 2015), they exhibit specific traits and niche environmental preferences (Nichols et al., 2013; Ghamkhar et al., 2015). Recently, the Ts ssp. *subterraneum* cv. Daliak and ssp. *yanninicum* cv. Yarloop were resequenced and surveyed for SVs using an optical mapping strategy (Yuan et al., 2018). That investigation of two genotypes revealed 31 regions with multiple SVs. Our analysis has revealed SNPs and CNVs across the genome which have given insight into intra-specific and intra-subspecific relationships. The phylogenetic analysis of sequence from 36 cultivars in this study described groups that reflect the known taxonomical relationships with Groups 1a, 1b and 2, which are comprised of Ts ssp. *subterraneum*, and Groups 3 and 4 made up of Ts ssp. *yanninicum* and Ts ssp. *brachycalycinum*, respectively. Splitting the Ts ssp. *subterraneum* accessions into three groups suggests that the molecular assessment of this subspecies contradicts or adds previously cryptic insights to the taxonomic classification. The cultivars reflect their known pedigree and taxonomic categorization apart from three exceptions. Cultivar Larisa was placed with the Ts ssp. *subterraneum* cultivars in Group 1a based on genome data yet is taxonomically categorized as Ts ssp. *yanninicum*. Similarly, genomic data indicates Mt Barker should be classified as Ts ssp. *yanninicum* rather than its taxonomic Ts ssp. *subterraneum* characterization. Woogenellup is a naturalized ecotype and considered to be Ts ssp. *subterraneum*, but the sequence data indicates it is part of Group 4, Ts ssp. *brachycalycinum.* The phylogenetic relationship of Woogenellup suggests similarities with the other groups, hinting at a possible hybridization event between Ts subspecies, most likely Ts ssp. *subterraneum* and Ts ssp. *brachycalycinum*, based on the genomic outcomes. It may also be a result of an admixture event in seed increase, as discussed below. Nevertheless, these discrepancies between genomic and taxonomic data, particularly the partitioning of Ts ssp. *subterraneum* across three groups suggest that the subspecific classification should be reviewed.

Assessment of SNP variants across pseudomolecules showed differences among the identified population groups with Group 2 being the most variable and Groups 3 and 4 the least. Spatial trends for the SNPs within each pseudomolecule were consistent with decreases at the telomeres and in many cases towards the center, consistent with metacentric chromosome structure in genus *Trifolium* (Williams et al., 2012). CNVs showed similar patterns although some regions of increased variation were found in different sites to the SNP variants and in some cases were specific to different Ts Groups. In summary, the detailed genetic diversity at the genome level captured in this study has provided valuable insight into chromosome-wide variant analysis and its relationship among and within Ts groupings.

Given the autogamous reproduction of Ts, an unexpected outcome was the sequence variation between multiple accessions of cultivars Coolamon, Denmark and Leura shown in the phylogenetic tree. The differences among multiple accessions of the same cultivar were greater than that of two very closely related cultivars, Geraldton and its mutant-derivative Uniwager. While the differences among accessions of the same cultivar could represent trapped heterozygosity, it also suggests admixture of two or more populations into an accession. This can happen during seed increase at either genebank or breeding company level as cross-contamination of seed lots is one of many issues faced by genebanks (Singh et al., 2012), but can be detected and corrected using next generation sequencing (Singh et al., 2019) even when phenotypic differences are cryptic. This was exemplified by extending our genomic survey to 10 individual plants within two accessions of cv Daliak and three of cv Woogenellup, revealed a likely case of admixture in accession Woogenellup AK1342. Admixture may also be a contributing factor to some accessions in the phylogenetic tree aligning with one group when pedigree data and conventional subspecies designation suggests placement in another group. For instance, the Woogenellup accession AK1342 with apparent admixture is assigned to Group 4 based on genomic data, but pedigree suggests affinity with members of Group 1. Given the challenges of admixture detection in outcrossing forage species including Tr, Tp and Ms; Ts may offer useful insight into frequency of accession mixtures within forage genebanks.

### Conclusions

This research has provided a new high-quality assembly of the Ts genome containing additional sequence data, *de novo* reassembly and pseudomolecule ordering. These data in conjunction with Hi-C scaffolding underpin significant improvements in assembly size and quality metrics. In addition to refining phylogenetic relationships with other economic forage legumes, this assembly has enabled new insight to relationships among and within Ts subspecies and cultivars. The discovery of likely admixture in accessions suggests molecular analysis of genebank accessions to provide definitive accession sources is warranted. Despite potential admixture, the distinct sub-grouping of cultivars into four groups, specifically Group 2 (Ts ssp. *subterraneum*) and its proximity to Groups 3 (Ts ssp. *yanninicum*) and 4 (Ts ssp. *brachycalicinum*) rather than Group 1 (Ts ssp. *subterraneum*), indicates a review of sub-specific classification in the species is justified.

Ts is of interest for multiple economically significant traits. It exhibits variation for suppression of methanogenesis in the rumen (Kaur et al., 2017a; Ghamkhar et al., 2018), which genome information can help further elucidate. Genome data may also offer insight into adaptive variation to edaphic stress in forage legumes. The new Ts assembly further enables comparative genetic analysis for key traits such as geocarpy with access to other geocarpic species such as *Arachis hypogaea* (Bertioli et al., 2019). Improving Ts for key traits including methane inhibition as a mitigating response to emissions from pastoral agriculture, and other traits of interest such as geocarpy is an important task for forage breeders. To enable acceleration of this work, assembling a Ts pangenome and identification of structural variants, including among known aneuploid sources, would provide an invaluable understanding of this species. This continued pangenome development should ideally include expression data for traits of interest and specifically climate adaptation traits among the Ts core collection. There is now an opportunity for application of this Ts genome and assembly in plant improvement in trait genetic analysis, genomic selection, improved cultivar and accession quality assurance and tracking, as well as utilization of genebank material.

## Data availability statement

The datasets presented in this study can be found in online repositories. The names of the repository/repositories and accession number(s) can be found in the text. The assembled genome and gene sequences are available at Plant GARDEN (https://plantgarden.jp/ja/list/t3900/genome/t3900.G002). The genome sequence data have been submitted to the DDBJ/ENA/NCBI public sequence databases under the BioProject ID PRJDB7187 (https://www.ncbi.nlm.nih.gov/bioproject/PRJDB7187/). Sequence data can also be found at the DNA Data Bank of Japan (DDBJ) Sequence Read Archive (https://www.ddbj.nig.ac.jp/ddbj/updt-form-e.html) under the following submission codes: DRA007081 (hirakawa-0130); DRA007082 (hirakawa-0129); DRA007083 (hirakawa-0128); DRA007084 (hirakawa-0131); DRA007085 (hirakawa-0132); DRA007086 (hirakawa-0133); DRA007087 (hirakawa-0135); DRA007088 (hirakawa-0136). Additional repositories for sequence data are detailed in **Supplementary Table S2**.

## Author contributions

KS: Data curation; Formal analysis; Investigation; Visualization; Writing – original draft. RM: Data curation; Formal analysis; Investigation; Visualization; Writing – original draft. HH: Data curation; Formal analysis; Investigation; Visualization; Writing – original draft. HN: Data curation; Formal analysis; Investigation; Visualization; Writing – original draft. AG: Data curation; Formal analysis; Investigation; Visualization; Writing – original draft. BB: Conceptualization; Writing – original draft; Writing – review & editing. KG: Conceptualization; Project administration; Resources; Supervision; Funding acquisition; Writing – original draft; Writing – review & editing. AGG: Conceptualization, Supervision; Visualization, Writing – original draft; Writing – review & editing. SI: Conceptualization; Data curation; Formal analysis; Funding acquisition; Investigation; Project administration; Resources; Supervision; Visualization; Writing – original draft; Writing – review & editing. All authors contributed to the article and approved the submitted version
