# Supplementary material for: An improved reference genome for Trifolium subterraneum L. provides insight into molecular diversity and intra-specific phylogeny
Source: Front Plant Sci. 2023 Feb 15;14:1103857. doi: 10.3389/fpls.2023.1103857 (PMC9975737; doi:10.3389/fpls.2023.1103857)
Supplement: Supplementary file 1 [file DataSheet_1.docx]

Supplementary Material

# Supplementary Figures and Tables

##
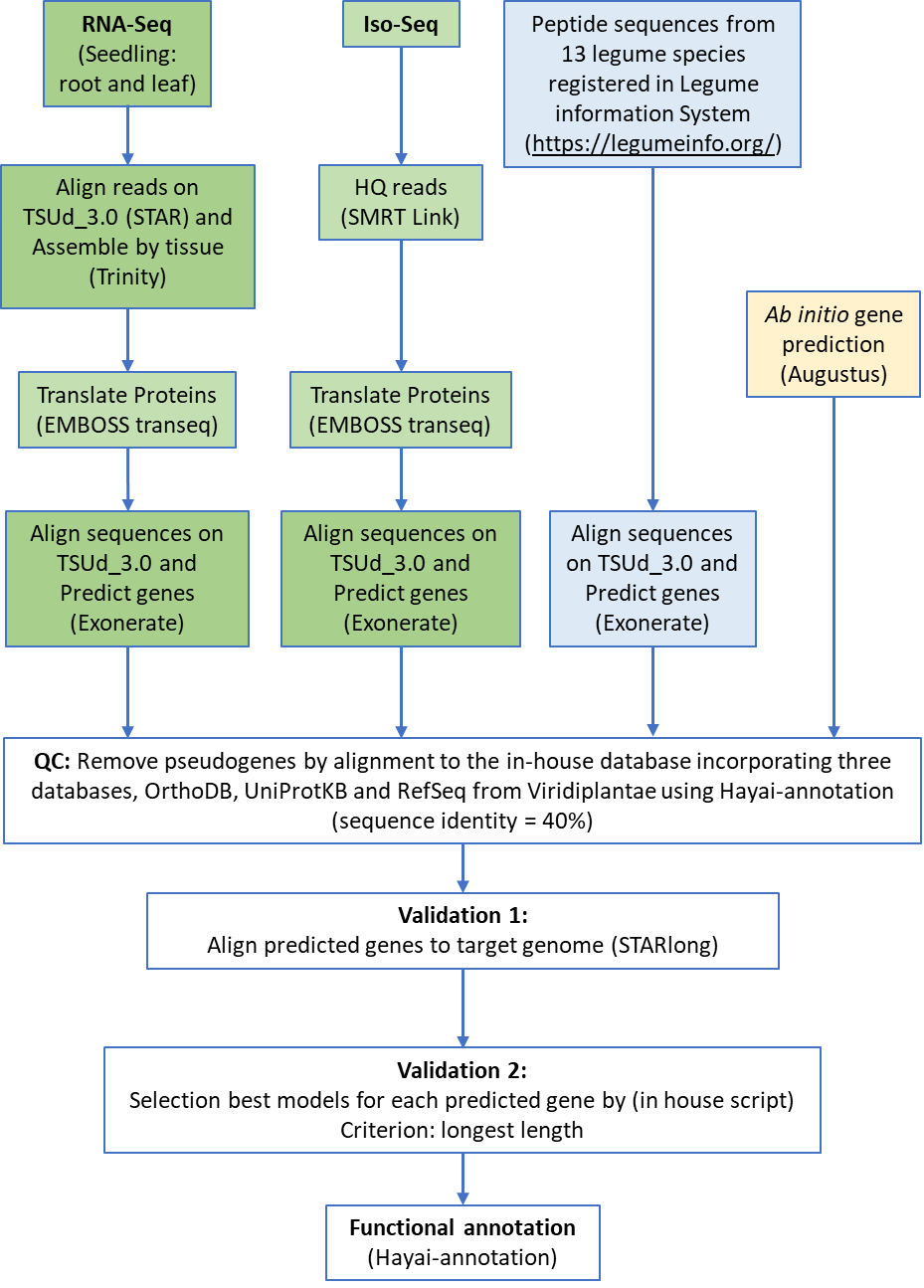
Supplementary Figures

**Supplementary Figure S1:** Gene prediction and functional annotation flowchart.


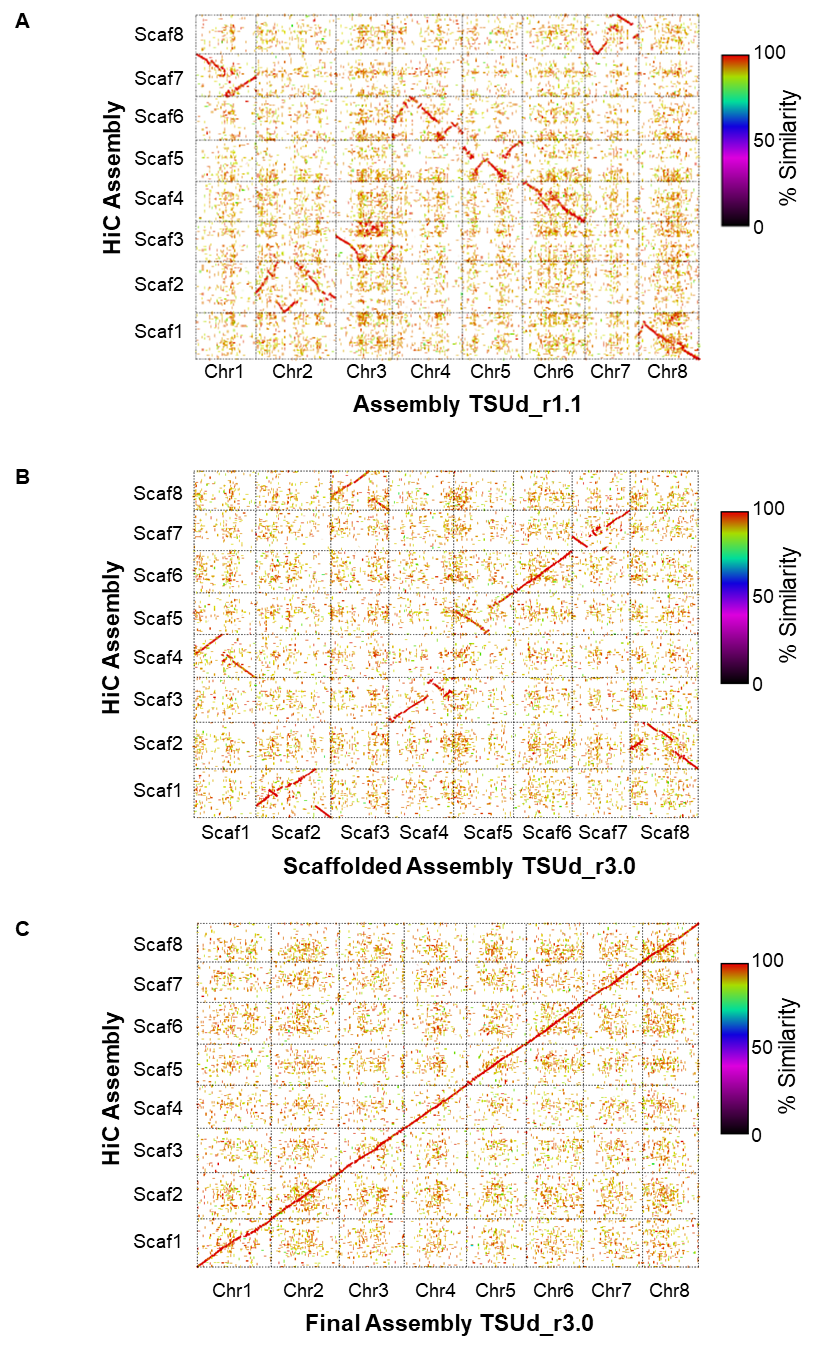
**Supplementary Figure S2:** NUCmer-derived (Kurtz, Phillippy et al. 2004) matrix plots showing synteny between a HiC assembly generated in this study and scaffolded or final assemblies of subterranean clover. **A)** synteny between HiC scaffolding based on short read assembly generated in this paper and the original assembly TSUd_r1.1 (Hirakawa, Kaur et al. 2016). **B)** The scaffolded TSUd_r3.0 assembly, incorporating short read, PacBio and TruSeq Synthetic Long-Read data, aligned with a HiC-derived scaffolding of the same data set. **C)** Alignment of HiC scaffolding with the final TSUd_r3.0 assembly which had been re-ordered based on the HiC analysis.


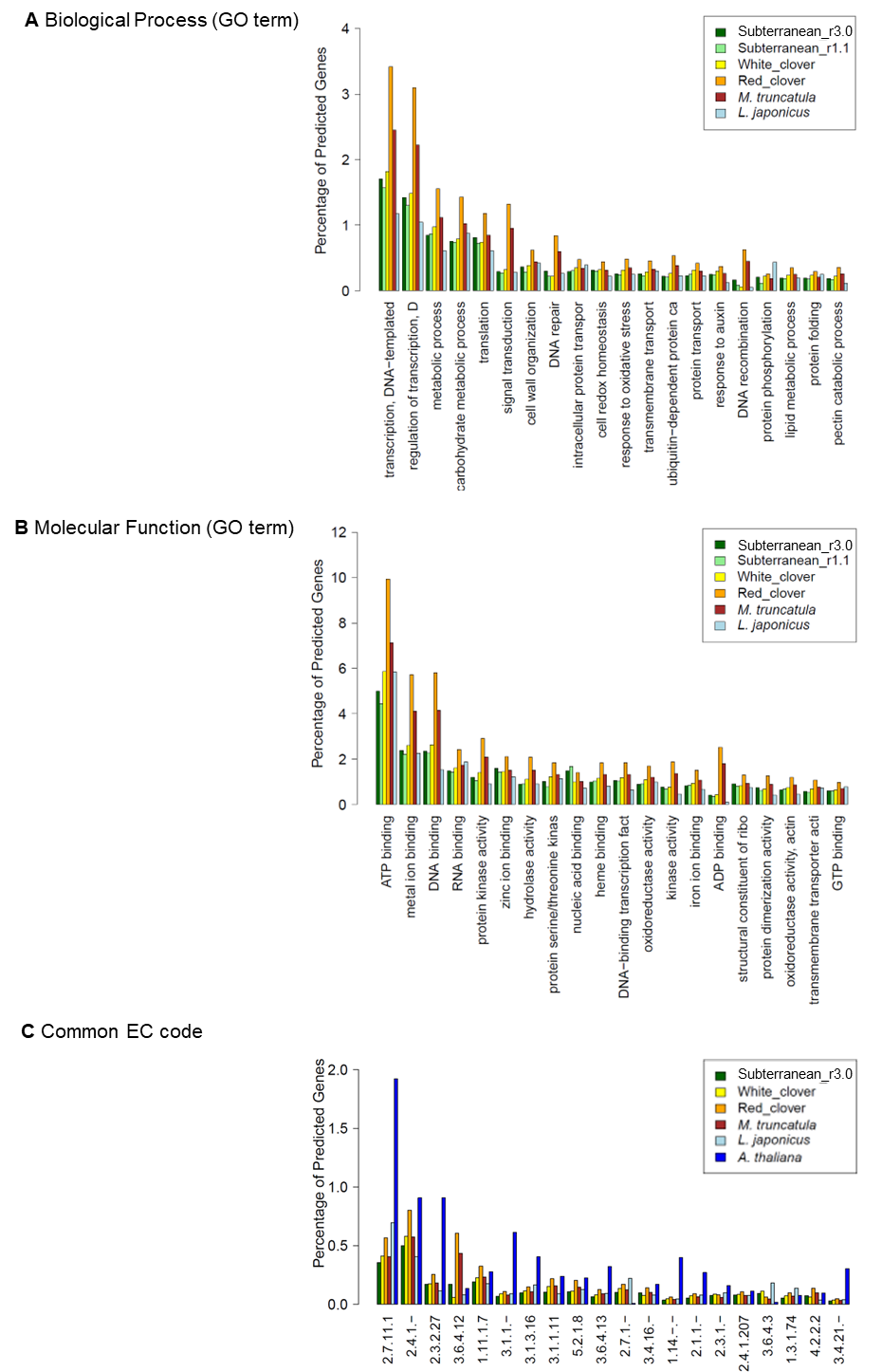
 **Supplementary Figure S3:** Distribution of molecular function based on gene annotation in the genomes of the first (TSUd_r1.1) and the latest (TSUd_r3.0) subterranean clover assembly, white clover (*T. repens*), red clover (*T. pratense*), *M. truncatula* and *Lotus japonicus*. **A)** Gene Ontology (GO) terms Biological Process. **B)** GO terms Molecular Function. **C)** Common Enzyme Commission (EC) numbers.


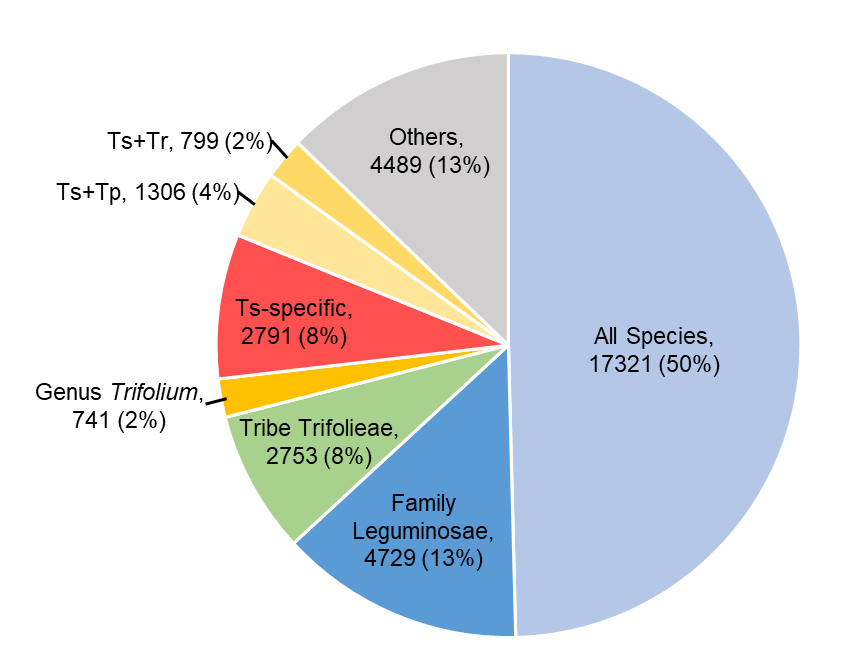


**Supplementary Figure S4:** The number and proportion of 41,979 predicted subterranean clover (Ts) genes clustered with those of other species comprising red clover (*Trifolium pratense*; Tp), white clover (*T. repens*; Tr); *Medicago truncatula* (Mt), *Lotus japonicus* (Lj) and *Arabidopsis thaliana* (At). Of the 41,979 Ts genes, 34,929 were in clusters and the remaining 7,050 were singletons. The groupings were: All Species (Ts+Tp+Tr+Mt+Lj+At); Family Leguminosae (Ts+Tp+Tr+Mt+Lj); Tribe Trifoliae (Ts+Tp+Tr+Mt); and Genus *Trifolium* (Ts+Tp+Tr); Ts-specific (unique to Ts); Others: comprised the remaining combinations of species such as At+Ts, At+Ts+Mt, Ts+Mt, Tr+At.


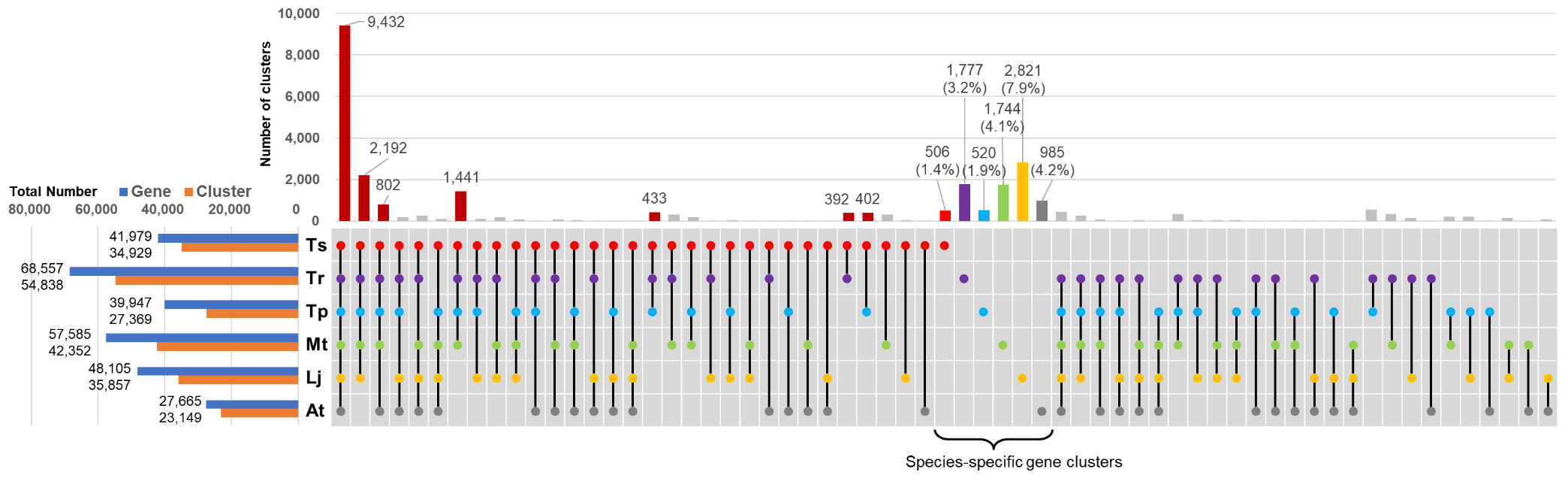


**Supplementary Figure S5:** An UpSet plot of the number of gene clusters derived from a comparative analysis of the predicted gene sets of subterranean clover (Ts), red clover (*Trifolium pratense*; Tp), white clover (*T. repens*; Tr); *Medicago truncatula* (Mt), *Lotus japonicus* (Lj) and Arabidopsis *thaliana* (At). There were 28,724 clusters across the six species and the plot shows the number of gene clusters in common for different species combinations. The focus is on Ts, where numbers of clusters in common <320 are grey. Species-specific clusters show those unique to each species and includes the proportion (%) of the total number of clusters for each species. The graph to the left of the plot shows the number clustered genes (blue) and number of gene clusters (red) for each species.


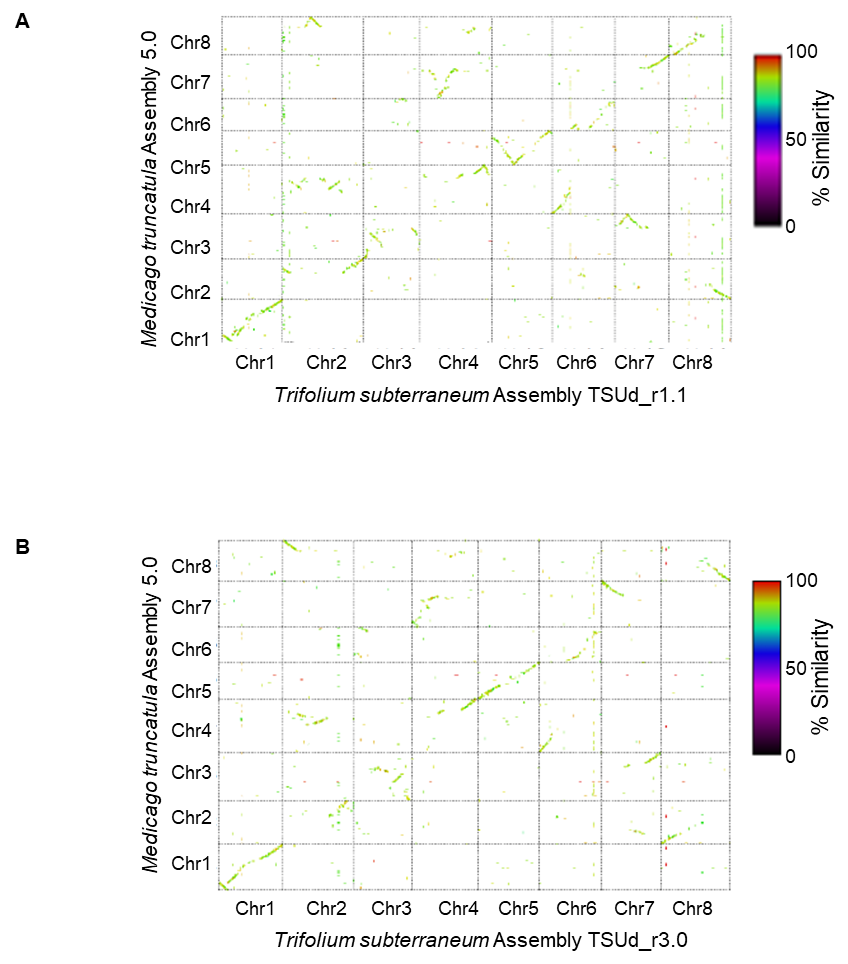


**Supplementary Figure S6:** NUCmer-derived (Kurtz, Phillippy et al. 2004) matrix plots showing synteny between *Medicago truncatula* reference genome v5.0 and **A)** the original subterranean clover assembly (TSUd_r1.1; (Hirakawa, Kaur et al. 2016)) and **B)** the latest assembly TSUd_r3.0.


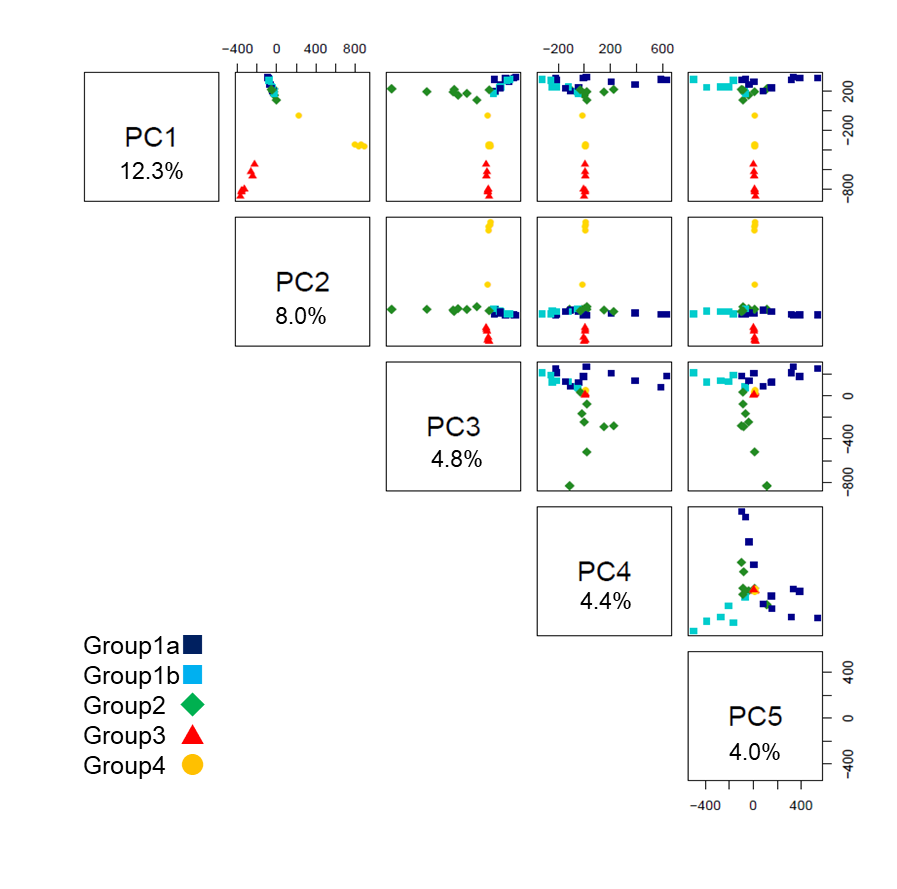


**Supplementary Figure S7:** Principal component analysis (PCA) plots across five components (PC1 – PC5) showing distribution of 36 subterranean clover cultivars based on diversity data from 7,789,537 single nucleotide polymorphism (SNPs). The groupings align with those described in **Figure 4**, and the values below the PCs indicate the proportion of variation explained by each principal component.

**
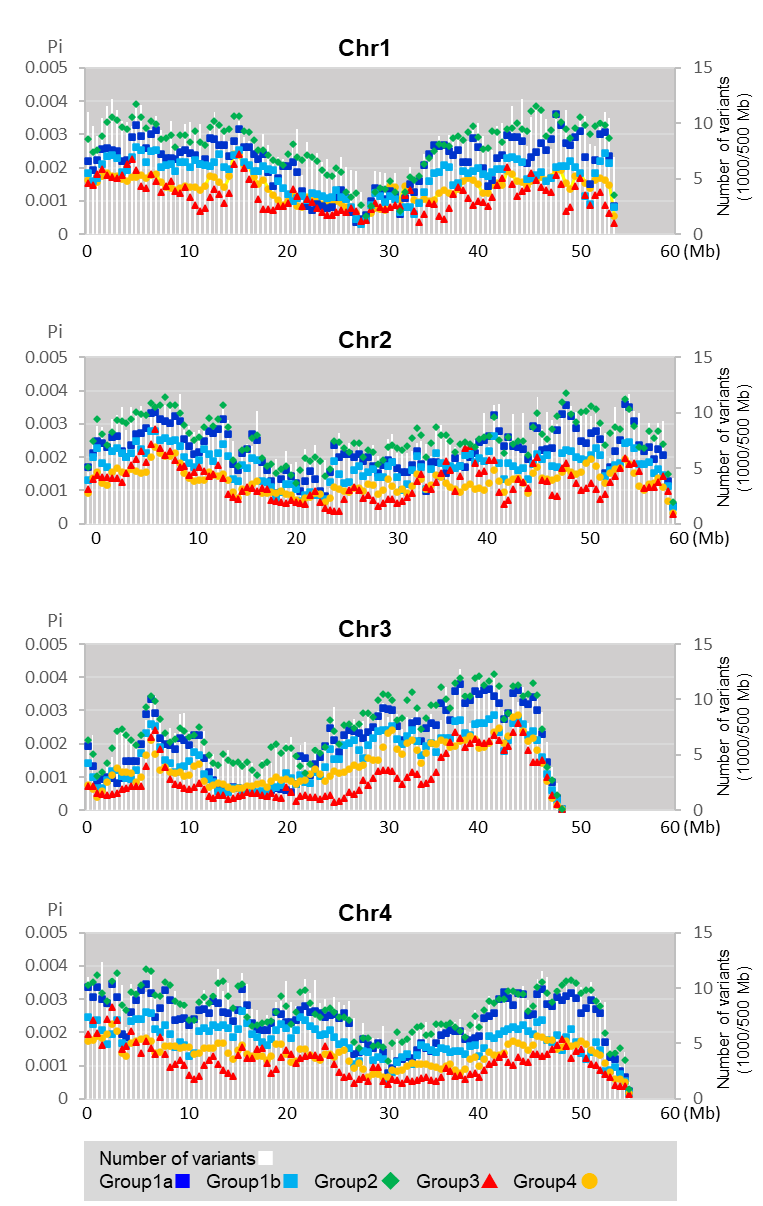
Supplementary Figure S8: A)** Distribution of number of variants (all) and the number of nucleotide differences per site between two sequences in a 500 kb window within a population (Pi) for each subterranean clover group or subgroup as defined in Figure 4. Chromosomes (Chr) 1 to 4.

**
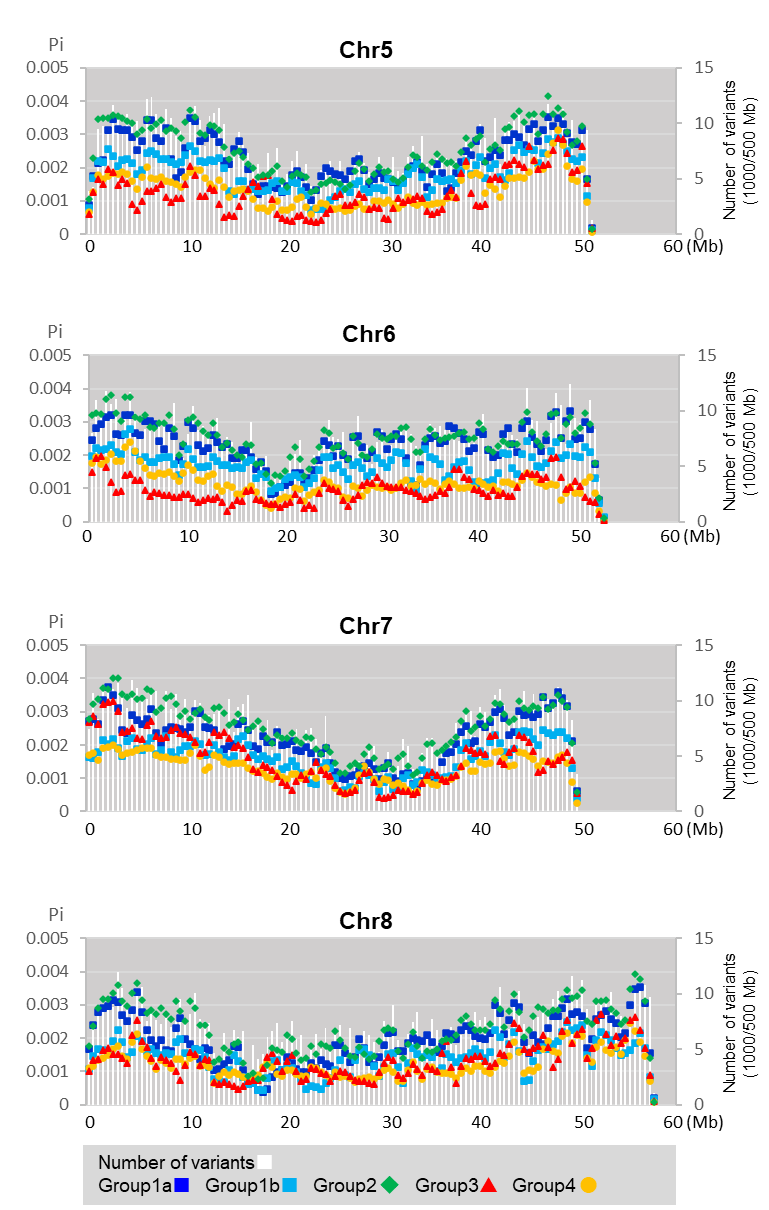
Supplementary Figure S8: B)** Distribution of number of variants (all) and the number of nucleotide differences per site between two sequences in a 500 kb window within a population (Pi) for each subterranean clover group or subgroup as defined in Figure 4. Chromosomes (Chr) 5 to 8.


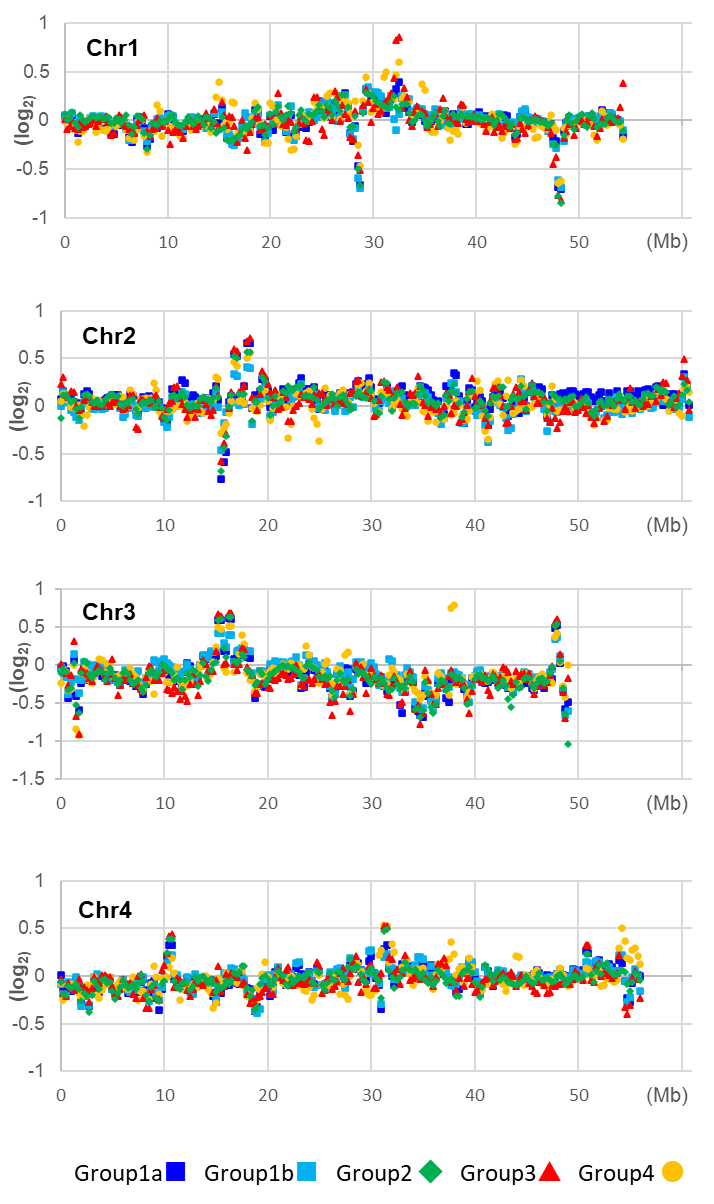
**Supplementary Figure S9:** **A)** Distribution of mean copy number variants (CNV) of each group or subgroup (Figure 4) in 500 kb window variants across the subterranean clover chromosomes (Chr) 1 to 4 based on sequence data from 39 individuals representing 36 subterranean clover cultivars.


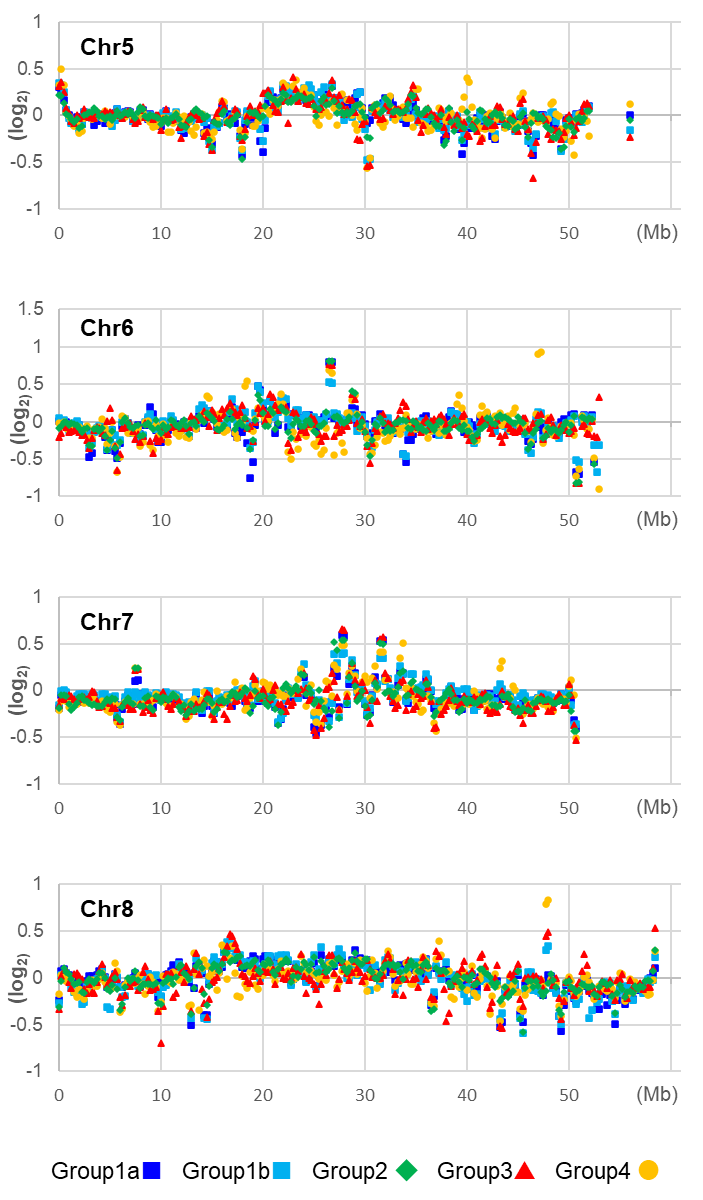
**Supplementary Figure S9:** **B)** Distribution of mean copy number variants (CNV) of each group or subgroup (Figure 4) in 500 kb window variants across the subterranean clover chromosomes (Chr) 5 to 8 based on sequence data from 39 individuals representing 36 subterranean clover cultivars.


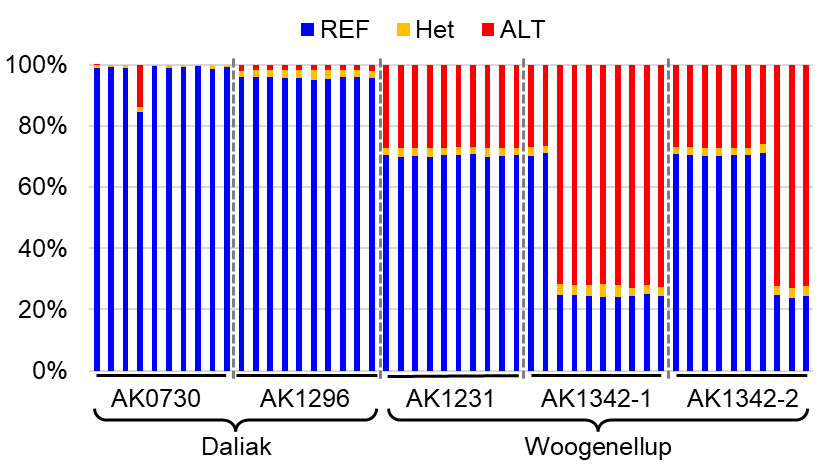


**Supplementary Figure S10:** Percentage of genotypes identified using 4,141 single nucleotide polymorphism (SNP) variants based on dd-RAD-Seq in individual plants from different seed lots of subterranean cultivars ‘Daliak’ and ‘Woogenellup’. Missing data were excluded from the calculations. The AK nomenclature are identifiers for seed lots stored in Margot Forde Forage Germplasm Centre located at AgResearch, Palmerston North, New Zealand. REF, Het and ALT were reference, heterozygous and alternative genotypes, respectively, relative to the TSUd_r3.0 assembly.

## Supplementary Tables

Supplementary Table S1: Statistics of sequencing libraries used to provide data for subterranean clover genome assembly TSUd_r3.0. Length represents either the length of every read in the raw output from the Illumina instruments, or the average length per read in the case of other platforms. Coverage represents the total base coverage given an estimated genome size of 544 Mb, and not the fragment coverage from pair-end and mate-pair libraries.

| Library | Instrument | Type | Read Length (nt) | Insert | No. Reads | Total Bases (Gb) | Coverage |
| --- | --- | --- | --- | --- | --- | --- | --- |
| Previous^1^ | Range of Hi/MiSeq /Roche 454 | SE, PE, MP | 97 -301 | 0 – 20 Kb | 2801 M | 171.7 | 316x |
| ***Illumina*** |  |  |  |  |  |  |  |
| TSLR^2^ |  | SE | ~3,255 | NA | 1146 M | 2.8 | 5x |
| ***PacBio*** |  |  |  |  |  |  |  |
| M160206^2^ | PacBio RS-II | SE | ~10,672 | NA | 477,975 | 5.6 | 10x |
| **Total** |  |  |  |  |  | **180.1** | **331x** |

^1^Hirakawa, Kaur et al. 2016.

^2^Generated in this project. Supplementary Table 2. The TSLR 106.6 Gb of sequence data resolved to 2.8 Gb of data after assembly into the synthetic long reads, which were then added to the genome assembly process.

SE=single-end; PE=paired-end; MP=mate-paired; TSLR=Illumina TruSeq Synthetic Long-Read sequence; NA=not applicable.

**Following page:**

**Supplementary Table S2:** DNA and transcript sequence data and repositories for cv Daliak used in this study, and sequence details and data repositories for the 38 additional re-sequenced Ts cultivar accessions.

| **Accession^a^** | **Sequence** | **Library** | **Platform** | **Read length (nt)** | **Number of reads** | **Total bases** | **Accession #^b^** |
| --- | --- | --- | --- | --- | --- | --- | --- |
| Daliak | Genome | Illumina Synthetic Long Reads | HiSeq 2000 | 93 | 1,146,554,124 | 106,629,533,532 | DRX135771-DRX134777, DRX135823 |
| Daliak | Genome | Single | PacBio RS-II | 10,672 (mean) | 477,975 (Sub reads) | 5,100,906,147 (Sub reads) | DRX135779 |
| Daliak | Genome | Hi-C-derived Illumina Paired-end | NextSeq 500 | 80 | 211,051,434 | 16,884,114,720 | DRX135778 |
| Daliak | Transcripts (leaves, roots, seedlings) | Illumina Paired-end | MiSeq | 301 | 80733700 | 24,300,843,700 | DRX135780-DRX135783 |
| Daliak | Transcripts (seedlings) | Single | PacBio Sequel | 15,853 (mean) | 1,058,762 (CCS)^d^ | 16,784,090,418 (CCS) | DRX135822 |
| Antas (AK1298) | AK1298 | Illumina Paired-end | HiSeq X | 151 | 99,748,794 | 15,062,067,894 | DRR144469 |
| BacchusMarsh (AK0518) | AK0518 | Illumina Paired-end | HiSeq X | 151 | 100,422,748 | 15,163,834,948 | DRR144448 |
| Bindoon (AK1363) | AK1363 | Illumina Paired-end | HiSeq X | 151 | 97,701,396 | 14,752,910,796 | DRR144473 |
| Campeda (AK1359) | AK1359 | Illumina Paired-end | HiSeq X | 151 | 93,169,126 | 14,068,538,026 | DRR144471 |
| Clare (AK0516) | AK0516 | Illumina Paired-end | HiSeq X | 151 | 99,739,246 | 15,060,626,146 | DRR144447 |
| Coolamen (AK1338) | AK1338 | Illumina Paired-end | HiSeq2000 | 101 | 104,671,290 | 9,734,429,970 | DRR144438 |
| Coolamon (AK1358) | AK1358 | Illumina Paired-end | HiSeq X | 151 | 105,215,690 | 15,887,569,190 | DRR144470 |
| Dalkeith (AK0725) | AK0725 | Illumina Paired-end | HiSeq X | 151 | 108,290,598 | 16,351,880,298 | DRR144454 |
| Denmark (AK1006) | AK1006 | Illumina Paired-end | HiSeq X | 151 | 95,516,728 | 14,423,025,928 | DRR144464 |
| Denmark (AK1343) | AK1343 | Illumina Paired-end | HiSeq2000 | 101 | 89,181,792 | 8,293,906,656 | DRR144443 |
| Dinninup (AK0521) | AK0521 | Illumina Paired-end | HiSeq X | 151 | 113,956,088 | 17,207,369,288 | DRR144449 |
| Dwalganup (AK0606) | AK0606 | Illumina Paired-end | HiSeq X | 151 | 119,221,218 | 18,002,403,918 | DRR144450 |
| Enfield (AK1235) | AK1235 | Illumina Paired-end | HiSeq X | 151 | 110,376,676 | 16,666,878,076 | DRR144466 |
| Geraldton (AK0727) | AK0727 | Illumina Paired-end | HiSeq X | 151 | 110,760,756 | 16,724,874,156 | DRR144456 |
| Gosse (AK1005) | AK1005 | Illumina Paired-end | HiSeq X | 151 | 100,068,120 | 15,110,286,120 | DRR144463 |
| Goulburn (AK1285) | AK1285 | Illumina Paired-end | HiSeq X | 151 | 87,402,834 | 13,197,827,934 | DRR144467 |
| GreenRange (AK1221) | AK1221 | Illumina Paired-end | HiSeq X | 151 | 98,064,970 | 14,807,810,470 | DRR144465 |
| Junee (AK0703) | AK0703 | Illumina Paired-end | HiSeq X | 151 | 101,236,102 | 15,286,651,402 | DRR144453 |
| Karridale (AK0996) | AK0996 | Illumina Paired-end | HiSeq X | 151 | 102,885,730 | 15,535,745,230 | DRR144460 |
| Larisa (AK679) | AK679 | Illumina Paired-end | HiSeq2000 | 101 | 92,227,802 | 8,577,185,586 | DRR144445 |
| Leura (AK1008) | AK1008 | Illumina Paired-end | HiSeq2000 | 101 | 95,706,572 | 8,900,711,196 | DRR144436 |
| Leura (AK1340) | AK1340 | Illumina Paired-end | HiSeq2000 | 101 | 84,701,594 | 7,877,248,242 | DRR144440 |
| Meteora (AK0699) | AK0699 | Illumina Paired-end | HiSeq X | 151 | 111,988,556 | 16,910,271,956 | DRR144452 |
| Monti (AK1297) | AK1297 | Illumina Paired-end | HiSeq X | 151 | 116,221,648 | 17,549,468,848 | DRR144468 |
| Mt Barker (AK674) | AK674 | Illumina Paired-end | HiSeq2000 | 101 | 101,138,446 | 9,405,875,478 | DRR144444 |
| Nangeela (AK0345) | AK0345 | Illumina Paired-end | HiSeq X | 151 | 119,646,840 | 18,066,672,840 | DRR144446 |
| Napier (AK1341) | AK1341 | Illumina Paired-end | HiSeq2000 | 101 | 98,978,010 | 9,204,954,930 | DRR144441 |
| Northam (AK0728) | AK0728 | Illumina Paired-end | HiSeq X | 151 | 103,734,870 | 15,663,965,370 | DRR144457 |
| Nuba (AK0808) | AK0808 | Illumina Paired-end | HiSeq X | 151 | 85,842,648 | 12,962,239,848 | DRR144459 |
| Nungarin (AK0729) | AK0729 | Illumina Paired-end | HiSeq X | 151 | 113,639,260 | 17,159,528,260 | DRR144458 |
| Rosabrook (AK1360) | AK1360 | Illumina Paired-end | HiSeq X | 151 | 103,678,514 | 15,655,455,614 | DRR144472 |
| Rosedale (AK1004) | AK1004 | Illumina Paired-end | HiSeq X | 151 | 152,708,798 | 23,059,028,498 | DRR144462 |
| Seaton Park (AK1339) | AK1339 | Illumina Paired-end | HiSeq2000 | 101 | 96,154,428 | 8,942,361,804 | DRR144439 |
| Tallarook (AK1326) | AK1326 | Illumina Paired-end | HiSeq2000 | 101 | 106,226,856 | 9,879,097,608 | DRR144437 |
| Trikkala (AK0726) | AK0726 | Illumina Paired-end | HiSeq X | 151 | 117,579,086 | 17,754,441,986 | DRR144455 |
| Uniwager (AK0619) | AK0619 | Illumina Paired-end | HiSeq X | 151 | 115,187,644 | 17,393,334,244 | DRR144451 |
| Woogenellup (AK1342) | AK1342 | Illumina Paired-end | HiSeq2000 | 101 | 73,667,678 | 6,851,094,054 | DRR144442 |
| Yarloop (AK0997) | AK0997 | Illumina Paired-end | HiSeq X | 151 | 106,761,836 | 16,121,037,236 | DRR144461 |
| ^a^ Letters in parenthesis are accession IDs in Margot Forage Germplasm Center. | | | |  |  |  |  |
| ^b^ EXPERIMENT accession numbers registered in DDBJ Sequence Read Archive (DRA) - sequence repository. | | | | | |  |  |
| ^c^ Total reads from both ends of the pair-end sequenced fragments | | |  |  |  |  |  |
| ^d^ PacBio Consensus sequence | |  |  |  |  |  |  |
| Shaded accessions indicate cultivars with multiple accessions. | | |  |  |  |  |  |

**Following page:**

**Supplementary Table S3:** Numbers of called genotypes based on the 7,789,537 single nucleotide polymorphism (SNP) loci identified among the re-sequenced 39 accessions representing 36 cultivars.

| **Accession^a)^** | **Genotype call at each of 7,789,537 SNP loci.** | | | | **Genotype calls as a percentage of total (%)** | | | |
| --- | --- | --- | --- | --- | --- | --- | --- | --- |
|  | **Reference** | **Heterozygous** | **Alternative** | **Missing** | **Reference** | **Heterozygous** | **Alternative** | **Missing** |
| Antas (AK1298) Grp 4 | 3,467,890 | 240,640 | 2,210,461 | 1,870,166 | 44.5 | 3.1 | 28.4 | 24.0 |
| Bacchus Marsh (AK0518) Grp 1a | 5,209,003 | 186,608 | 1,682,947 | 710,599 | 66.9 | 2.4 | 21.6 | 9.1 |
| Bindoon (AK1363) Grp 1b | 4,851,343 | 781,669 | 1,328,079 | 828,066 | 62.3 | 10.0 | 17.0 | 10.6 |
| Campeda (AK1359) Grp 2 | 4,676,840 | 1,328,830 | 998,480 | 785,007 | 60.0 | 17.1 | 12.8 | 10.1 |
| Clare (AK0516) Grp 4 | 3,686,903 | 135,061 | 2,137,673 | 1,829,520 | 47.3 | 1.7 | 27.4 | 23.5 |
| Coolamon (AK1338) Grp 1a | 4,144,881 | 676,428 | 958,079 | 2,009,769 | 53.2 | 8.7 | 12.3 | 25.8 |
| Coolamon (AK1358) Grp 1a | 5,264,369 | 188,988 | 1,721,361 | 614,439 | 67.6 | 2.4 | 22.1 | 7.9 |
| Dalkeith (AK0725) Grp 2 | 5,484,138 | 154,569 | 1,472,925 | 677,525 | 70.4 | 2.0 | 18.9 | 8.7 |
| Denmark (AK1006) Grp 1b | 5,127,034 | 208,891 | 1,680,317 | 772,915 | 65.8 | 2.7 | 21.6 | 9.9 |
| Denmark (AK1343) Grp 1b | 3,616,045 | 621,348 | 813,633 | 2,738,131 | 46.4 | 8.0 | 10.4 | 35.2 |
| Dinninup (AK0521) Grp 1a | 5,206,300 | 186,042 | 1,816,027 | 580,788 | 66.8 | 2.4 | 23.3 | 7.5 |
| Dwalganup (AK0606) Grp 2 | 5,567,616 | 155,403 | 1,524,678 | 541,460 | 71.5 | 2.0 | 19.6 | 7.0 |
| Enfield (AK1235) Grp 1a | 4,830,946 | 985,312 | 1,218,685 | 754,214 | 62.0 | 12.6 | 15.6 | 9.7 |
| Geraldton (AK0727) Grp 2 | 5,451,146 | 171,180 | 1,605,262 | 561,569 | 70.0 | 2.2 | 20.6 | 7.2 |
| Gosse (AK1005) Grp 3 | 4,176,550 | 573,936 | 2,062,132 | 976,539 | 53.6 | 7.4 | 26.5 | 12.5 |
| Goulburn (AK1285) Grp 1b | 4,925,747 | 178,606 | 1,586,928 | 1,097,876 | 63.2 | 2.3 | 20.4 | 14.1 |
| GreenRange (AK1221) Grp 1a | 5,388,396 | 267,960 | 1,424,504 | 708,297 | 69.2 | 3.4 | 18.3 | 9.1 |
| Junee (AK0703) Grp 1a | 5,507,611 | 381,394 | 1,265,854 | 634,298 | 70.7 | 4.9 | 16.3 | 8.1 |
| Karridale (AK0996) Grp 1a | 5,172,734 | 225,617 | 1,756,075 | 634,731 | 66.4 | 2.9 | 22.5 | 8.1 |
| Larisa (AK679) Grp 1a | 3,592,204 | 316,464 | 927,542 | 2,952,947 | 46.1 | 4.1 | 11.9 | 37.9 |
| Leura (AK1008) Grp 1b | 3,734,019 | 179,689 | 1,080,374 | 2,795,075 | 47.9 | 2.3 | 13.9 | 35.9 |
| Leura (AK1340) Grp 1b | 3,232,146 | 293,998 | 842,581 | 3,420,432 | 41.5 | 3.8 | 10.8 | 43.9 |
| Meteora (AK0699) Grp 3 | 4,318,512 | 195,190 | 2,317,222 | 958,233 | 55.4 | 2.5 | 29.7 | 12.3 |
| Monti (AK1297) Grp 3 | 4,302,706 | 316,775 | 2,232,477 | 937,199 | 55.2 | 4.1 | 28.7 | 12.0 |
| MtBarker (AK674) Grp 3 | 3,373,593 | 199,886 | 1,614,141 | 2,601,537 | 43.3 | 2.6 | 20.7 | 33.4 |
| Nangeela (AK0345) Grp 1a | 5,255,173 | 178,497 | 1,790,436 | 565,051 | 67.5 | 2.3 | 23.0 | 7.3 |
| Napier (AK1341) Grp 3 | 3,101,774 | 195,775 | 1,425,010 | 3,066,598 | 39.8 | 2.5 | 18.3 | 39.4 |
| Northam (AK0728) Grp 2 | 4,841,035 | 1,627,233 | 772,632 | 548,257 | 62.1 | 20.9 | 9.9 | 7.0 |
| Nuba (AK0808) Grp 4 | 3,300,651 | 143,663 | 2,115,041 | 2,229,802 | 42.4 | 1.8 | 27.2 | 28.6 |
| Nungarin (AK0729) Grp 2 | 5,602,127 | 1,429,494 | 300,170 | 457,366 | 71.9 | 18.4 | 3.9 | 5.9 |
| Rosabrook (AK1360) Grp 1b | 4,750,103 | 1,450,884 | 1,081,564 | 506,606 | 61.0 | 18.6 | 13.9 | 6.5 |
| Rosedale (AK1004) Grp 4 | 4,022,049 | 130,088 | 2,365,426 | 1,271,594 | 51.6 | 1.7 | 30.4 | 16.3 |
| SeatonPark (AK1339) Grp 2 | 3,776,392 | 155,222 | 994,104 | 2,863,439 | 48.5 | 2.0 | 12.8 | 36.8 |
| Tallarook (AK1326) Grp 1a | 3,950,455 | 282,155 | 1,048,876 | 2,507,671 | 50.7 | 3.6 | 13.5 | 32.2 |
| Trikkala (AK0726) Grp 3 | 4,286,757 | 284,154 | 2,394,879 | 823,367 | 55.0 | 3.6 | 30.7 | 10.6 |
| Uniwager (AK0619) Grp 2 | 5,417,232 | 171,838 | 1,586,541 | 613,546 | 69.5 | 2.2 | 20.4 | 7.9 |
| Woogenellup (AK1342) Grp 4 | 1,438,069 | 999,393 | 301,752 | 5,049,943 | 18.5 | 12.8 | 3.9 | 64.8 |
| Yarloop (AK0997) Grp 3 | 4,313,619 | 180,090 | 2,114,338 | 1,181,110 | 55.4 | 2.3 | 27.1 | 15.2 |
| Daliak Grp 2 | 6,880,270 | 93,507 | 23,119 | 792,261 | 88.3 | 1.2 | 0.3 | 10.2 |
| ^a)^ Letters in parenthesis are accession IDs in Margot Forage Germplasm Centre. Shaded rows identify multiple accessions of a cultivar. | | | |  |  |  |  |  |

## Supplementary References

Hirakawa, H., P. Kaur, K. Shirasawa, P. Nichols, S. Nagano, R. Appels, W. Erskine and S. N. Isobe (2016). "Draft genome sequence of subterranean clover, a reference for genus Trifolium." Scientific Reports **6**: 30358 (2016).

Kurtz, S., A. Phillippy, A. L. Delcher, M. Smoot, M. Shumway, C. Antonescu and S. L. Salzberg (2004). "Versatile and open software for comparing large genomes." Genome Biology **5**: R12 (2004).
